# Developmental convergence and divergence in human stem cell models of autism

**DOI:** 10.1038/s41586-025-10047-5

**Published:** 2026-01-29

**Authors:** Aaron Gordon, Se-Jin Yoon, Lucy K. Bicks, Jacqueline M. Martín, Greta Pintacuda, Stephanie Arteaga, Brie Wamsley, Qiuyu Guo, Lubayna Elahi, Ricardo E. Dolmetsch, Jonathan A. Bernstein, Ruth O’Hara, Joachim F. Hallmayer, Kasper Lage, Sergiu P. Pasca, Daniel H. Geschwind

**Affiliations:** 1 Program in Neurogenetics, Department of Neurology, David Geffen School of Medicine, University of California Los Angeles, Los Angeles, CA USA; 2 Department of Psychiatry and Behavioral Sciences, Stanford University, Stanford, CA USA; 3 Stanford Brain Organogenesis Program, Wu Tsai Neurosciences Institute and Bio-X, Stanford University, Stanford, CA USA; 4 Stanley Center for Psychiatric Research, Broad Institute, Cambridge, MA USA; 5 Tempero Bio, Boston, MA USA; 6 Department of Pediatrics, Stanford University, Stanford, CA USA; 7 Novo Nordisk Foundation Center for Genomic Mechanisms of Disease, Broad Institute of MIT and Harvard, Cambridge, MA USA; 8 Department of Human Genetics, David Geffen School of Medicine, University of California Los Angeles, Los Angeles, CA USA; 9 Program in Neurobehavioral Genetics, Semel Institute, David Geffen School of Medicine, University of California Los Angeles, Los Angeles, CA USA; 10 Center for Autism Research and Treatment, Semel Institute, David Geffen School of Medicine, University of California Los Angeles, Los Angeles, CA USA

**Keywords:** Disease model, Cellular neuroscience

## Abstract

Two decades of genetic studies in autism spectrum disorder (ASD) have identified more than 100 genes harbouring rare risk mutations^1–13^. Despite this substantial heterogeneity, transcriptomic and epigenetic analyses have identified convergent patterns of dysregulation across the ASD postmortem brain^14,15–17^. To identify shared and distinct mechanisms of ASD-linked mutations, we assembled a large patient collection of human induced pluripotent stem (hiPS) cells, consisting of 70 hiPS cell lines after stringent quality control representing 8 ASD-associated mutations, idiopathic ASD, and 20 lines from non-affected control individuals. Here we used these hiPS cell lines to generate human cortical organoids, profiling by RNA sequencing at four distinct time points up to 100 days after in vitro differentiation. Early time points harboured the largest mutation-specific changes, but different mutations converged on shared transcriptional changes as development progressed. We identified a shared RNA and protein interaction network, which was enriched in ASD risk genes and predicted to drive the observed downstream changes in gene expression. CRISPR–Cas9 screening of these candidate transcriptional regulators in induced human neural progenitors validated their downstream convergent molecular effects. These data illustrate how risk associated with genetically defined forms of ASD can propagate by means of transcriptional regulation to affect convergently dysregulated pathways, providing new insight into the convergent impact of ASD genetic risk on human neurodevelopment.

## Abstract (web summary)

Risk associated with genetically defined forms of autism spectrum disorder (ASD) can propagate by means of transcriptional regulation to affect convergently dysregulated pathways, providing insight into the convergent impact of ASD genetic risk on human neurodevelopment.

## Main

Autism spectrum disorder (ASD) is a common neurodevelopmental disorder (NDD), with a childhood prevalence of close to 2%^18^. The last decade of genetic studies has yielded hundreds of risk genes, consistent with extraordinary aetiological heterogeneity^1–4^. More than 100 high-confidence mutations have been associated with ASD in genetic studies^2,5–13^. These rare, usually de novo mutations with large effect sizes are expected to account for 10–15% of ASD cases^19,20^, whereas common genetic variation is predicted to explain at least 50% of genetic risk^21–23^. Overall, ASD shows a complex genetic architecture, with a substantial component derived from a collection of distinct, rare disorders with overlapping clinical features.

Despite its genetic heterogeneity, postmortem transcriptome analysis has revealed consistent changes in most individuals with idiopathic ASD, as well as individuals with a specific syndromic form of ASD, (dup)15q11–13 (refs. ^14,15–17^). However, the mechanisms by which distinct mutations can lead to convergent molecular pathology, and whether convergence occurs across rare forms of ASD remains unknown. Understanding how these processes develop is complicated by the fact that the expression of most ASD risk genes peaks during fetal development, yet gene expression studies in human brain are conducted after this critical developmental window has ended^16,24–27^. Several lines of evidence, including genetic^28^, genomic^29,30^, neuroimaging^31^ and neuropathology^32^, indicate that early neurodevelopment has an essential role in the development of ASD.

The advent of stem cell-based in vitro systems enables high-fidelity modelling of human brain development in NDDs^33–41^. Most studies have investigated relatively small numbers of lines from individuals with idiopathic ASD^42^, or focused on individual mutations, which have demonstrated the utility of human induced pluripotent stem (hiPS) cell-based systems to study the impact of ASD genetic risk on neurodevelopment^43–46^. In addition, recent work has highlighted the power of studying many genes in parallel, identifying evidence for molecular convergence using CRISPR-based perturbations of ASD risk genes on control genetic backgrounds^47–49^. However, studies of lines derived from affected individuals are a major gap in the field.

Here we profile a large cohort of hiPS cell lines, starting from 96 hiPS cell lines ascertained from individuals with 8 different mutations associated with ASD and 11 individuals with idiopathic ASD and 30 lines derived from 25 matched control participants. From each of these lines, we derived neural organoids using a guided differentiation approach to create human cortical organoids (hCOs)^50,51^. Using orthogonal analytic approaches, we find evidence for convergence during early neuronal differentiation in those with genetically defined forms, but no significant shared signal across the idiopathic cases. We identify and characterize a downregulated, chromatin and transcriptional network that contains several ASD risk genes, including members of the SWI–SNF complex. This network is predicted to drive the observed changes in downstream gene expression associated with these ASD susceptibility mutations. We use CRISPRi to validate the effects of many putative network drivers on downstream gene expression, which includes downregulation of important neurodevelopmental pathways including many ASD susceptibility genes.

## Generation of hCOs from hiPS cell lines

**Fig. 1:**
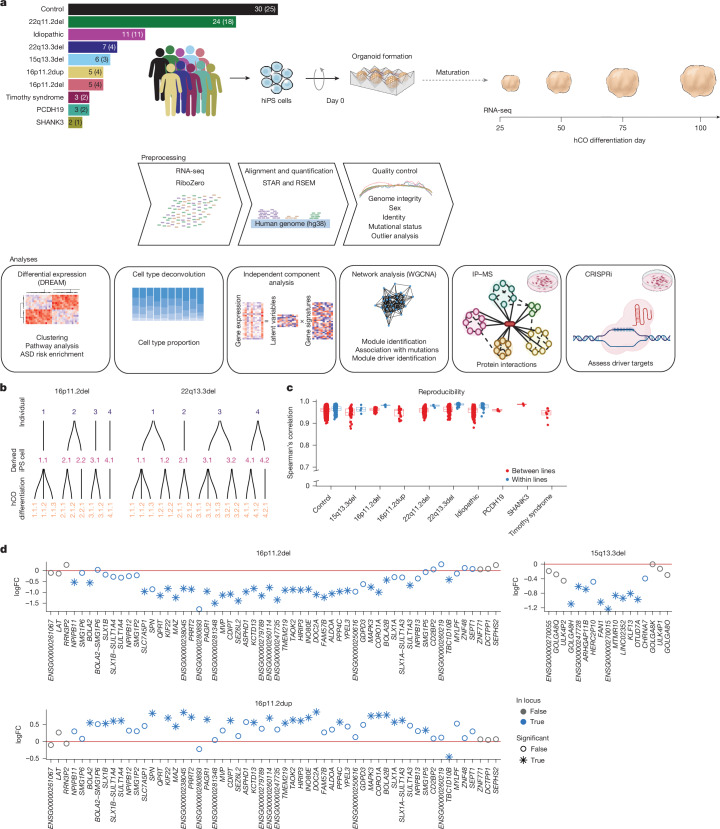
Experimental workflow and validation. **a**, Schematic workflow going from hiPS cells to cortical organoids to sequencing data. The number of hiPS cell lines and individuals (in parentheses) for each form of ASD and controls is indicated. **b**, Schematic representation of hCO differentiations derived from each hiPS cell line for two forms of ASD (16p11.2 deletion and 22q13.3 deletion). The other forms of ASD can be found in Extended Data Fig. 1. **c**, Spearman’s correlation of gene expression between samples from the same time point and form of ASD that were derived either from different lines (red) or from the same line (blue). The sample sizes for each group (number of differentiations) are as follows: day 25: control lines, *n* = 46; 15q13.3del, *n* = 7; 16p11.2del, *n* = 5; 16p11.2dup, *n* = 4; 22q11.2del, *n* = 13; 22q13.3del, *n* = 11; idiopathic, *n* = 12; PCDH19, *n* = 2; SHANK3, *n* = 2; and Timothy syndrome, *n* = 3. Day 50: control lines, *n *= 53; 15q13.3del, *n* = 7; 16p11.2del, *n* = 6; 16p11.2dup, *n* = 4; 22q11.2del, *n* = 15; 22q13.3del, *n* = 12; idiopathic, *n* = 15; *PCDH19*, *n* = 2; SHANK3, *n* = 2; and Timothy syndrome, *n* = 3. Day 75: control lines, *n* = 54; 15q13.3del, *n* = 7; 16p11.2del, *n* = 6; 16p11.2dup, *n* = 4; 22q11.2del, *n* = 23; 22q13.3del, *n* = 12; idiopathic, *n* = 15; PCDH19, *n* = 2; SHANK3, *n* = 2; and Timothy syndrome, *n* = 2. Day 100: control lines, *n* = 50; 15q13.3del, *n* = 6; 16p11.2del, *n* = 6; 16p11.2dup, *n* = 4; 22q11.2del, *n* = 15; 22q13.3del, *n* = 12; idiopathic, *n* = 15; PCDH19, *n* = 2; SHANK3, *n* = 2; and Timothy syndrome, *n* = 2. Boxplots show: centre, median; lower hinge, 25% quantile; upper hinge, 75% quantile; lower whisker, smallest observation greater than or equal to lower hinge −1.5× interquartile range; upper whisker, largest observation less than or equal to upper hinge +1.5× interquartile range. **d**, Genes within the CNVs are downregulated in deletions and upregulated in duplications, as exemplified by 16p11.2 deletion (del) and duplication (dup) and 15q13.3 deletion. 16p11.2del, 61.5% of genes; 16p11.2dup, 50% of genes and 15q13.3del, 52.9% of genes. *dreamlet *P* values of less than 0.005. Top illustration adapted from ref. ^51^, Springer Nature America; illustrations in the IP–MS and CRISPRi panels created in BioRender. Geschwind, D. (2025) (https://biorender.com/m2jkj03).

**Extended Data Fig. 1:**
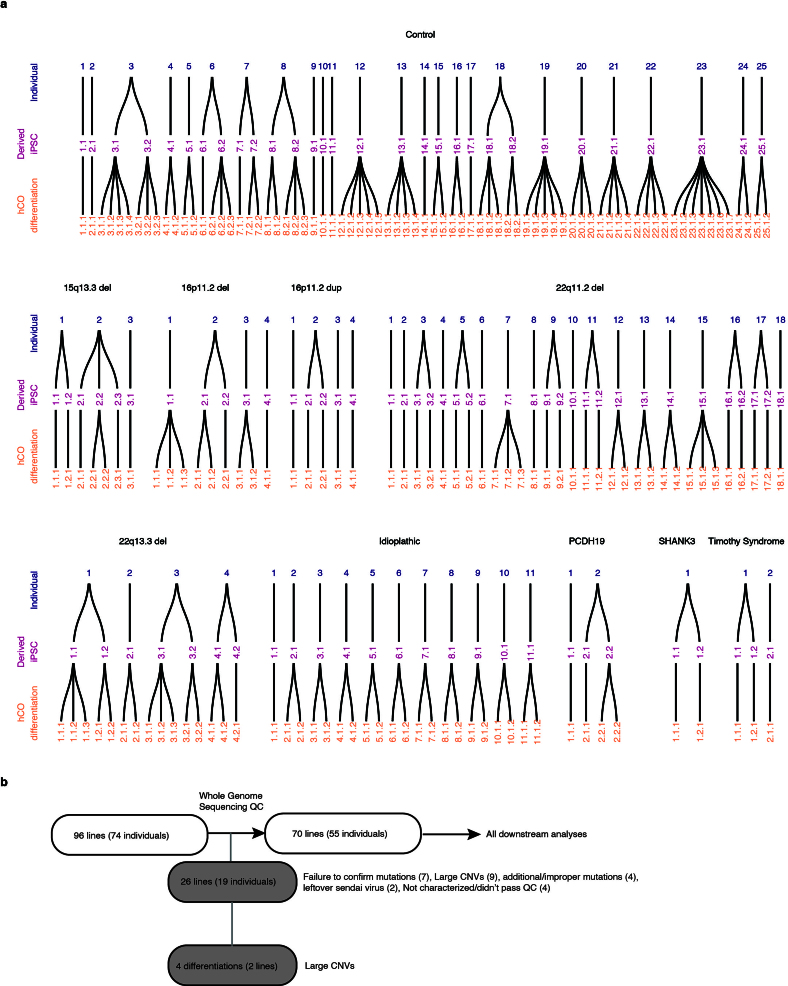
Experimental design. (**a**) Schematic representation of hCO differentiations from each derived hiPS cell line from each individual. (**b**) Schema of dropped lines due to quality control metrics.

We reprogrammed somatic cells (Methods) to generate hiPS cells from a cohort of individuals with eight different mutations associated with ASD: (1) 22q11.2 deletion, a 1.5–3-megabase (Mb) deletion that leads to a constellation of variably present symptoms including heart defects, craniofacial features, intellectual disability, ASD (roughly 20%) and psychosis (roughly 25%)^52,53^ (*n* = 18)^44^; (2) 22q13.3 deletion, known as Phelan–McDermid syndrome, a deletion spanning 130 kilobases (kb) to 9 Mb that includes *SHANK3*, among other genes and presents with developmental delay, hypotonia and impaired social interactions^54^ (*n *= 4); (3) 15q13.3 deletion, a 1.5-Mb deletion that presents variably with intellectual disability and epilepsy^55^ (*n* = 3); (4) 16p11.2 deletion, a roughly 600-kb deletion presenting variably with intellectual disability, motor impairments, communication deficits and ASD^56^ (*n* = 4); (5) 16p11.2 duplication, which can share intellectual disability and ASD phenotypes with the reciprocal deletion^57^ (*n* = 4); (6) Timothy syndrome, which is characterized by variants within the *CACNA1C* gene and presents with syndactyly, prolonged QT interval, ASD and intellectual disability^58,59^ (*n* = 2)^45,60,61^; (7) PCDH19-related disorder, which is associated with epilepsy and can also include intellectual disability and ASD^62,63^ (*n* = 2) and (8) the *SHANK3* R522W mutation (*n* = 1), a point mutation associated with neurodevelopmental risk^64^; mutations in *SHANK3* share behavioural phenotypes with the larger 22q13.3 deletion^61,65,66^. We also profiled individuals with idiopathic ASD with no known pathogenic variants (*n* = 11) and unaffected individuals (*n* = 25) (Fig. 1a and Supplementary Table 1) for a total of 74 individuals. For several individuals we used several hiPS cell lines to assess reproducibility, amounting to a total of 96 hiPS cell lines (Fig. 1a,b and Extended Data Fig. 1).

**Extended Data Fig. 2:**
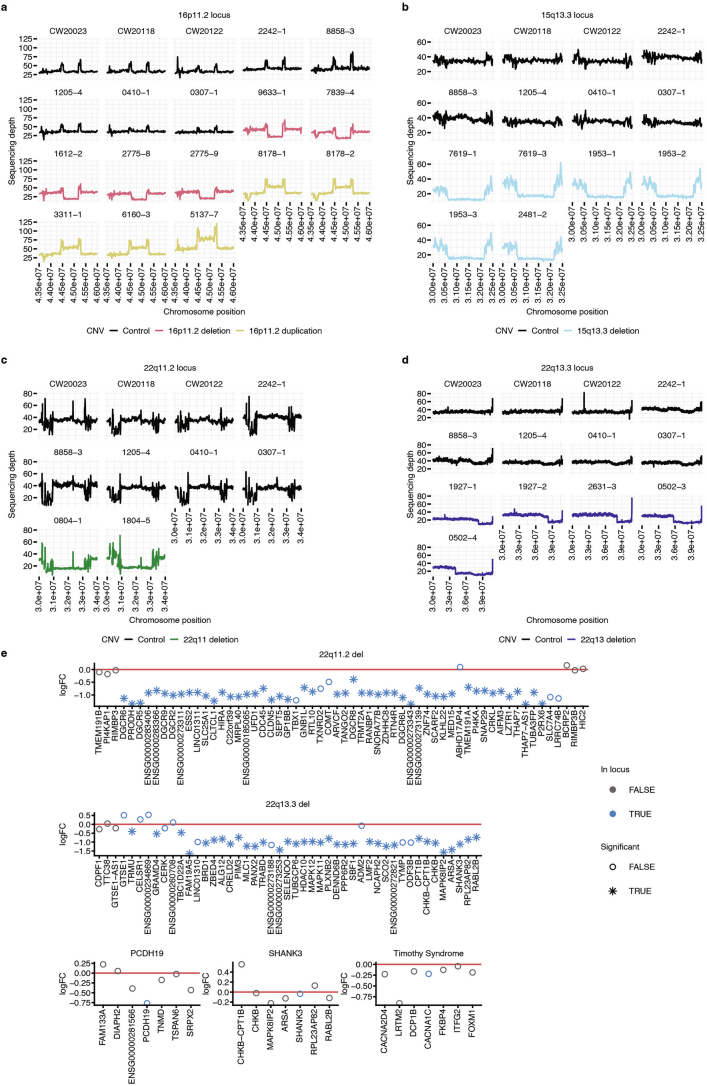
Sequencing depth in CNV loci. Whole genome sequencing depth in (**a**) 16p11.2 (**b**) 15q13.3 (**c**) 22q11.2 and (**d**) 22q13.3 loci averaged over 10 kb windows. (**e**) Mutational forms not shown in main figure showing genes inside the CNV locus or genes carrying the point mutations (marked in blue). Genes significantly differentially expressed over all time points are denoted by asterisks (dreamlet p-values of p < 0.005). The percent of DE genes in the CNV across the 4 time points is as follows: 22q13del - 77.3% of genes, 22q11del - 88.9% of genes.

**Extended Data Fig. 3:**
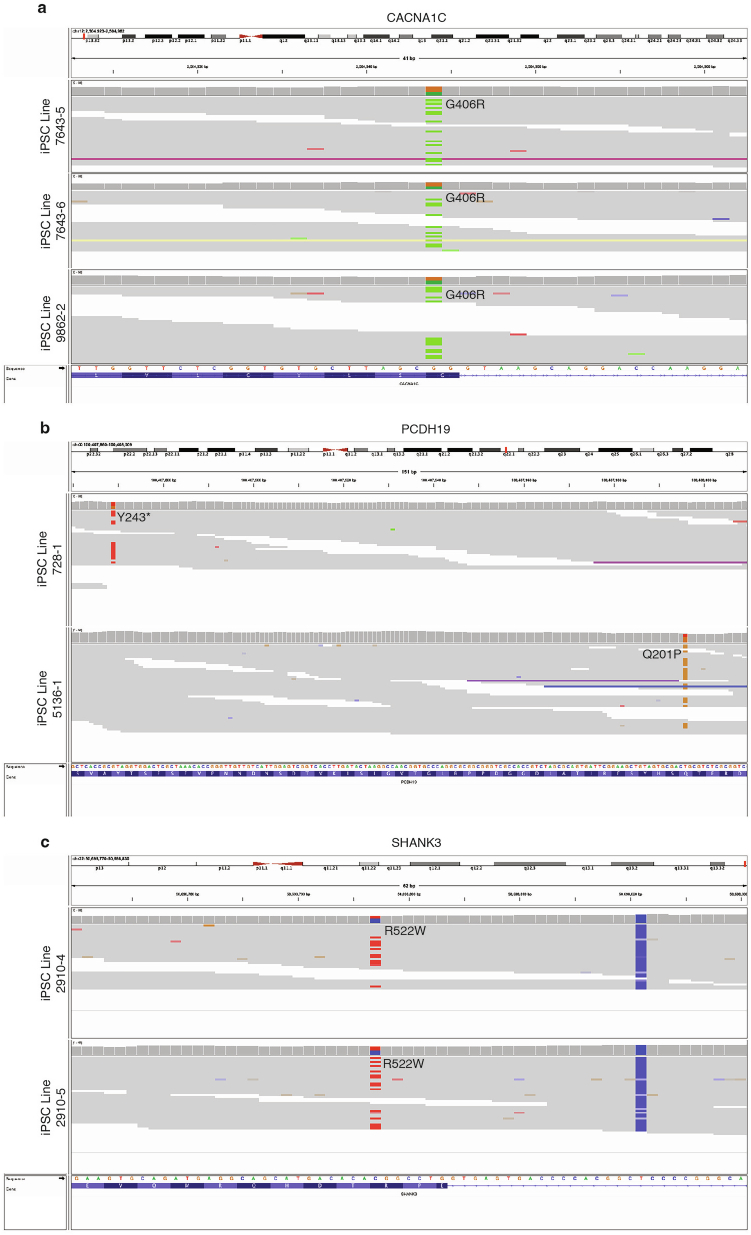
Point mutations identified using whole genome sequencing. (**a**) Point mutations in CACNA1C (G406R) identified in the 3 lines from individuals with Timothy syndrome. (**b**) Point mutations in PCDH19 (Y243* and Q201P) identified in the 2 lines from individuals with PCDH19 syndrome. (**c**) Point mutations in SHANK3 (R522W) identified in the 2 lines from the individual with SHANK3 mutation. All mutations were visualized with the Integrative Genomics Viewer (IGV).

We differentiated hiPS cell lines into hCOs for 100 days^51^, which yields hCOs that contain developing radial glia and ultimately give rise to functional glutamatergic neurons in a process that closely resembles cerebral cortical development^36,51^. We performed 172 unique hCO differentiations of the 96 lines (replicates for 49% of lines; control *n* = 75, idiopathic ASD *n* = 21, 22q11.2 deletion *n* = 31, 22q13.3 deletion *n* = 15, 15q13.3 deletion *n* = 7, 16p11.2 deletion* n* = 9, 16p11.2 duplication *n* = 5, Timothy syndrome *n* = 3, PCDH19 mutation *n* = 4 and SHANK3 mutation *n* = 2). We performed whole-genome sequencing and removed any lines with large copy number variations (CNVs), viral integration or in which the mutation of interest was absent or further mutations were present, which yielded 70 verified lines from 55 individuals passing quality control for downstream functional genomic analyses (Methods, Extended Data Figs. 1–3 and Supplementary Table 2).

We next analysed the transcriptomes of pooled hCOs at days 25, 50, 75 and 100 of hCO differentiation (Fig. 1a,b and Extended Data Fig. 1), which provides a quantitative, unbiased metric^67,68^. Day 25 corresponds to an early period in cortical development^36,50^, consisting primarily of cycling progenitors and radial glia^50,69^. As differentiation progresses, the cell composition evolves to consist of intermediate progenitors, deep layer postmitotic neurons and astroglial lineage cells^44,50^. After stringent RNA sequencing (RNA-seq) quality control and outlier analysis to remove low-quality samples (Fig. 1a, Extended Data Figs. 1–3 and Methods), 464 samples from 55 individuals (70 lines) were retained (Supplementary Table 1).

**Extended Data Fig. 4:**
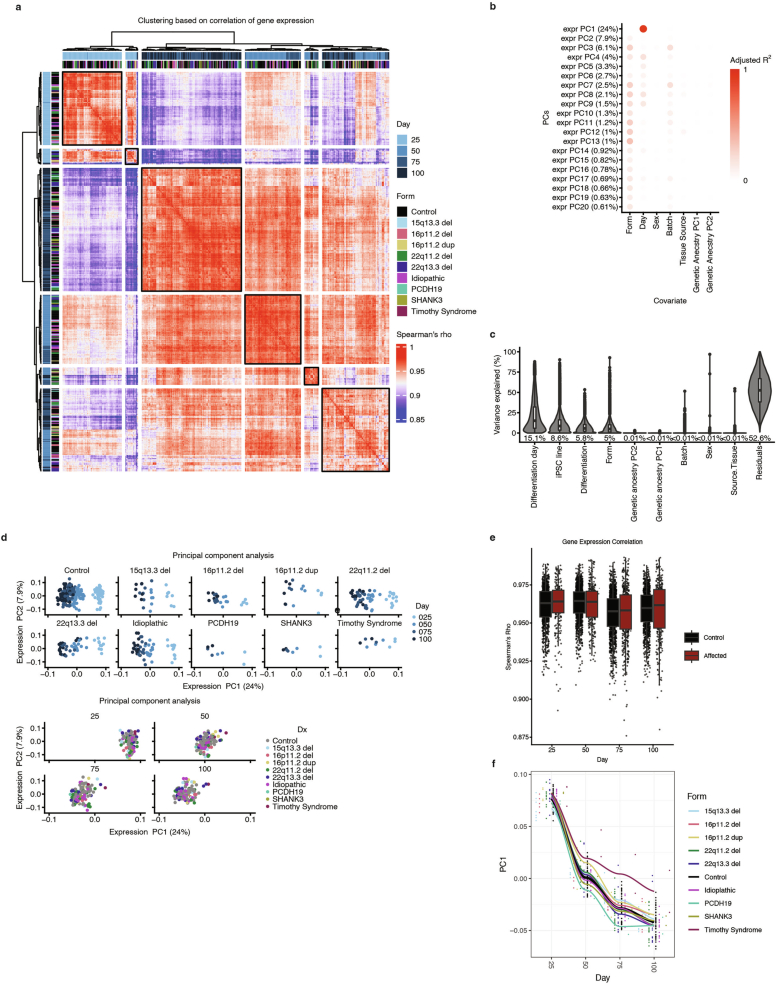
Sources of variation in the gene expression data. (**a**) Hierarchical clustering of all samples based on gene expression showing that samples cluster by differentiation day and the genetic form of ASD. Annotation colour bars represent day (shades of blue) and form (colourful). (**b**) Association of the top 20 gene expression principal components (PC) with the different covariates. Numbers in brackets on the y-axis are the percent of variance explained by these PCs. Differentiation time is highly associated with the 1^st^ PC (Adjusted R^2^ = 0.92). (**c**) Variance explained by each of the covariates for each gene (n = 17,963 genes). Distributions represent the density of the percent of variance explained. The median value of percent of explained variance for each variable are denoted below the plots. Boxplots in c and e show: centre, median, lower hinge – 25% quantile, upper hinge – 75% quantile, lower whisker – smallest observation greater than or equal to lower hinge –1.5× interquartile range, upper whisker – largest observation less than or equal to upper hinge +1.5× interquartile range. (**d**) Scatter plot of the first two gene expression PCs from each form of ASD. Colour represents the differentiation day (top) or form (bottom). PC1 explains 24% of the variation and is highly associated with differentiation day. PC2 explains 7.9% of the variation. Sample numbers: Day 25: Controls – 46, 15q13.3del – 7, 16p11.2del – 5, 16p11.2dup – 4, 22q11.2del – 13, 22q13.3del – 11, Idiopathic – 12, PCDH19 – 2, SHANK3 – 2, Timothy Syndrome – 3. Day 50: Controls – 53, 15q13.3del – 6, 16p11.2del – 6, 16p11.2dup – 4, 22q11.2del – 15, 22q13.3del – 12, Idiopathic – 15, PCDH19 – 2, SHANK3 – 2, Timothy Syndrome – 3. Day 75: Controls – 54, 15q13.3del – 7, 16p11.2del – 6, 16p11.2dup – 4, 22q11.2del – 23, 22q13.3del – 12, Idiopathic – 15, PCDH19 – 2, SHANK3 – 2, Timothy Syndrome – 2. Day 100: Controls – 50, 15q13.3del – 6, 16p11.2del – 6, 16p11.2dup – 4, 22q11.2del – 15, 22q13.3del – 12, Idiopathic – 15, PCDH19 – 2, SHANK3 – 2, Timothy Syndrome – 2. (**e**) Correlation of gene expression with control samples and forms (Affected) shows no difference in within-control and within-form correlation across development. Correlation by age shows a slight effect (Anova Eta2 = 0.04) with a slight decrease in correlation across development. Total number of pair-wise correlations: Day 25: Control – 1035, Affected – 241, Day 50: Control – 1378, Affected – 317, Day 75: Control – 1431, Affected – 469, Day 100: Control – 1225, Affected – 315. (**f**) Pseudotime testing of forms using linear mixed model and contrasts with Wald test shows no significant FDR corrected differences in pseudotime (PC-1).

We observed high reproducibility both between (overall mean Spearman correlation 0.96, range 0.88–0.99) and within (overall mean Spearman correlation 0.97, range 0.92–0.98) individuals, similar to published metrics^36,44,51^ (Fig. 1c and Methods). The largest driver of variation was stage of differentiation (Extended Data Fig. 4). Genes within the CNV boundaries were downregulated in the deletion carriers and upregulated in the duplication carriers as expected (Fig. 1d and Extended Data Fig. 2). Although several of the genes affected by point mutations showed a trend towards downregulation (*PCDH19*, log fold change (logFC) of −0.77, false discovery rate (FDR) of 0.45; *CACNA1C*: logFC = −0.22, FDR = 0.78), these changes were not significant, as expected^70–72^ (Extended Data Figs. 2 and 3).

## Transcriptomic relationships across forms and time

**Fig. 2:**
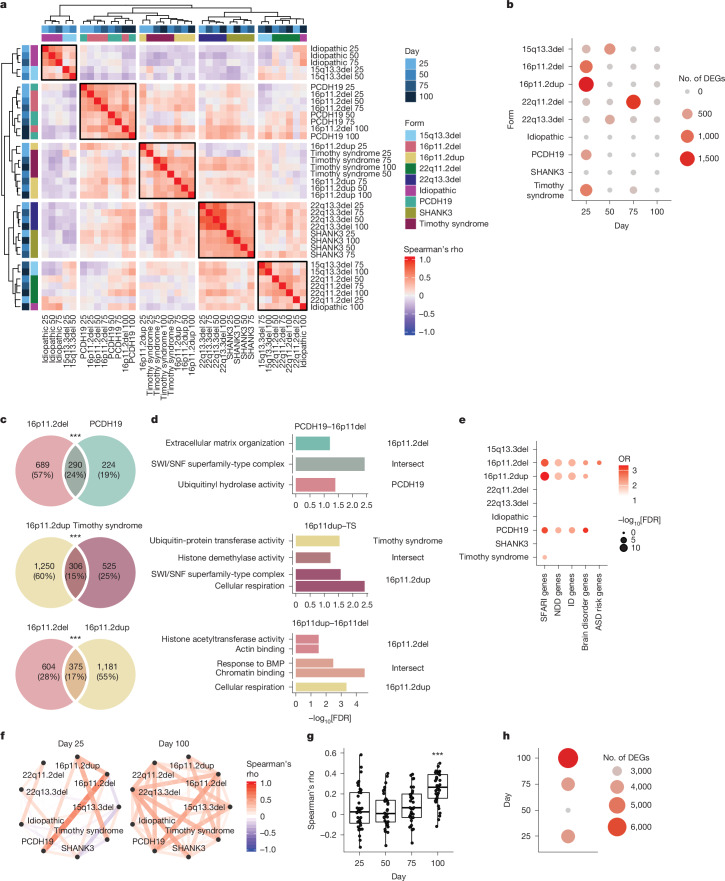
Clustering and overlap of ASD forms points to increasing convergence across development. **a**, Heatmap of hierarchical clustering based on correlation of logFC between different conditions (ASD form and differentiation day). Annotation bars (shades of blue) represent differentiation day and colourful boxes represent ASD form. **b**, The number of DEGs in each ASD form during hCO differentiation. Colours and sizes of the circles represent the number of DEGs. **c**, Overlap in DEGs between forms of ASD at day 25. Number and percentage of total genes are indicated. Statistics were derived from Fisher’s exact test (two-sided). ***FDR < 0.005. **d**, Gene ontology terms significantly enriched in unique and intersecting DEGs. Statistics were derived from clusterProfiler enrichGO. **e**, Overlaps between DEGs at day 25 and ASD^2,8^, NDD and intellectual disability risk genes^91^. Colour represents OR and size represents −log_10_FDR. Only positive significant overlaps (OR > 1 and FDR < 0.05) are shown. Statistics were derived from Fisher’s exact test (two-sided). **f**, Network representation of the correlation (Spearman’s rho) between logFC of different ASD forms at days 25 and 100. Colour corresponds to rho value and line thickness corresponds to the rho absolute value. **g**, All correlations (Spearman’s rho) between logFC of the different forms. ANOVA followed by posthoc Tukey’s HSD; ANOVA *F*_3,140_ = 10.97, *P* = 1.6 × 10^−6^, Tukey HSD: day 100 to day 25 *P* = 2.67 × 10^−5^, day 100 to day 50 *P* = 8 × 10^−6^, day 100 to day 75 *P* = 7 × 10^−4^. Boxplots show: centre, median; lower hinge, 25% quantile; upper hinge, 75% quantile; lower whisker, smallest observation greater than or equal to lower hinge −1.5× interquartile range; upper whisker, largest observation less than or equal to upper hinge +1.5× interquartile range. Statistics were derived from *n* = 36 correlations per time point. ***Tukey HSD for all contrasts less than 0.001. **h**, The number of differentially expressed meta-significant genes comparing all affected forms with control participants. Colour and size of the circle represent the number of DEGs. BMP, bone morphogenetic protein; ID, intellectual disability.

**Extended Data Fig. 5:**
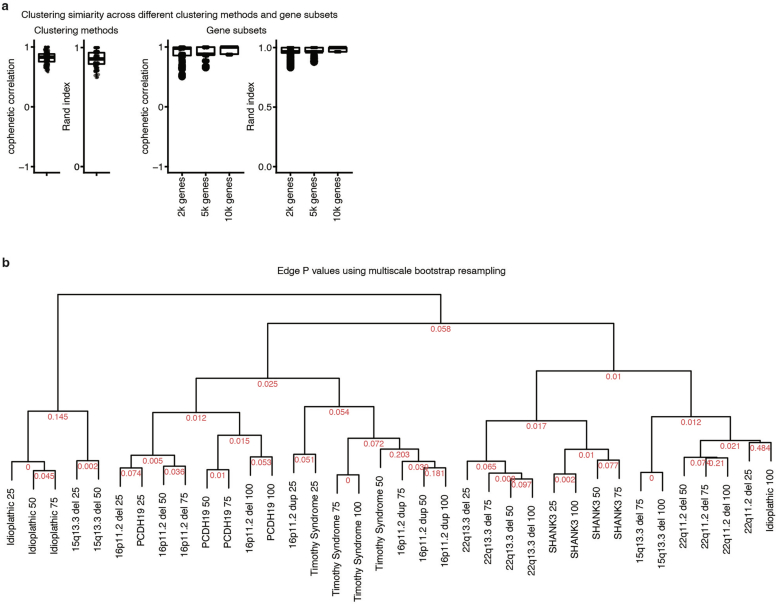
Validation of clusters based on correlation of logFC. (**a**) Cophenetic correlation and Rand index across multiple clustering and distance methods (left) and gene subsampling (right). Cophenetic correlation measures how faithfully the pairwise distances of the unmodeled data are preserved in the dendrogram. Rand index measures the similarity between two different clusterings of the same data. Number of individual correlations plotted: Clustering methods – 190. Gene subsets – 4950. Boxplots show: centre, median, lower hinge – 25% quantile, upper hinge – 75% quantile, lower whisker – smallest observation greater than or equal to lower hinge –1.5× interquartile range, upper whisker – largest observation less than or equal to upper hinge +1.5× interquartile range. (**b**) Cluster significance using multiscale bootstrap resampling. P values are indicated in red for each branching point. Bootstrap probability measures the frequency that a cluster appears in bootstrap replicates.

We observed several reliable, distinct, gene-expression clusters across forms (Methods and Supplementary Table 3), with participants harbouring the 16p11.2 deletion clustering with those with PCDH19-related disorder, 16p11.2 duplication clustering with Timothy syndrome and 22q13.3 and SHANK3 mutation clustering together (Fig. 2a). We performed bootstrapping to show that these clusters were robust (Extended Data Fig. 5 and Methods). Despite the strong effect of differentiation day on transcriptional profiles, most samples from individual genetic mutations clustered together across time points, showing a consistent effect of mutation over developmental progression (Fig. 2a).

**Extended Data Fig. 6:**
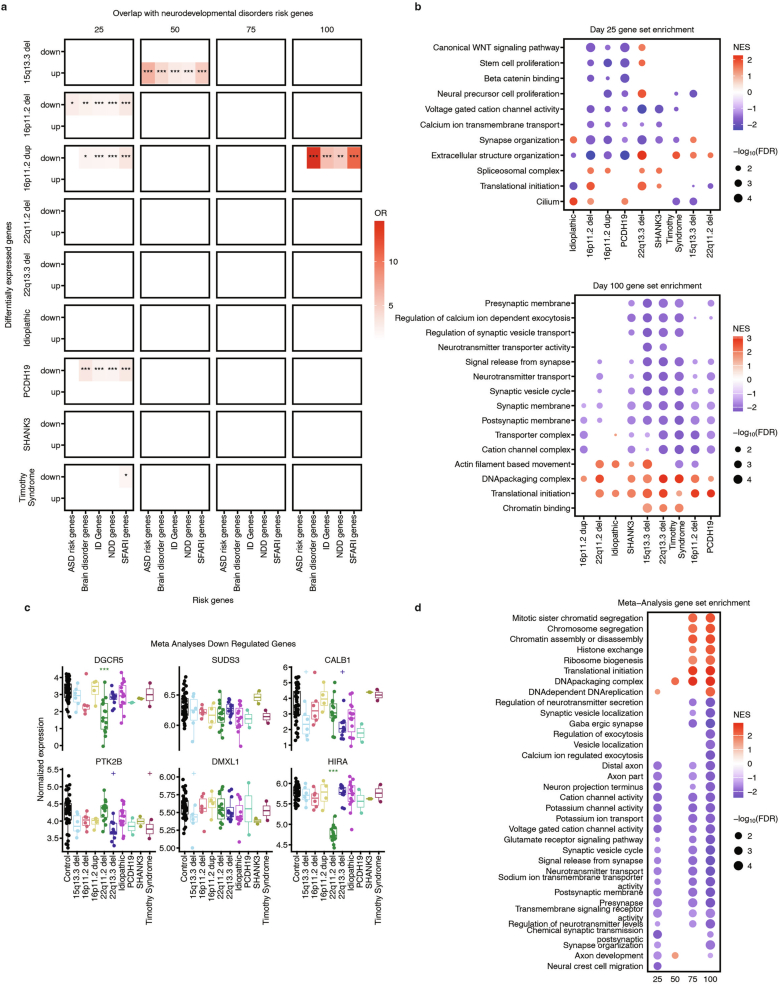
Annotation of differentially expressed genes. (**a**) Overlaps between differentially expressed genes at all time points and risk genes for ASD from either SFARI or from large scale whole exome (WES) sequencing studies^2,8^ as well as with neurodevelopmental disorders (NDDs) and intellectual disability (ID) risk genes^91^. Colour represents the OR (two-sided Fishers exact test) and the size of the point represented the -log_10_ FDR. Only positive significant overlaps (OR > 1 and FDR < 0.05) are shown. (**b**) Select gene ontology (GO) terms enriched in upregulated (red) or downregulated (blue) genes in each of the ASD forms at day 25 (top) or day 100 (bottom). Statistics derived from GSEA. (**c**) Distribution of normalized expression for meta analysis significant example genes at day 100. Boxplots show: centre, median, lower hinge – 25% quantile, upper hinge – 75% quantile, lower whisker – smallest observation greater than or equal to lower hinge –1.5× interquartile range, upper whisker – largest observation less than or equal to upper hinge +1.5× interquartile range. Number of samples (differentiations) for each form: Control – 50, 15q13.3del – 6, 16p11.2del – 6, 16p11.2dup – 4, 22q11.2del – 15, 22q13.3del – 12, Idiopathic – 15, PCDH19 – 2, SHANK3 – 2, Timothy Syndrome – 2. Asterisks denote dreamlet derived p-values: +p < 0.005,* FDR < 0.05, ** FDR < 0.01, *** FDR < 0.005. (**d**) Select GO terms enriched in upregulated or downregulated meta-analysis significant genes Colour corresponds to normalized enrichment score (NES). Point size corresponds to the level of significance (−log_10_(FDR)). Statistics derived from GSEA.

Next, we found that the largest changes in gene expression for individual forms were observed at early stages of hCO differentiation, with the largest number of differentially expressed genes (DEGs) found at day 25 in four forms (16p11.2 deletion, 979 genes; 16p11.2 duplication, 1,556 genes; PCDH19, 514 genes and Timothy syndrome, 832 genes), or at day 50 in 2 of the remaining forms (15q13.3 deletion, 616 genes and 22q13.3 deletion, 373 genes; Fig. 2b and Supplementary Table 3). Significant similarity was seen between genes differentially expressed at day 25 in samples harbouring deletion and duplication of 16p11.2 (375 out of 2,160 genes, *P* < 10^−16^, hypergeometric test) (Fig. 2c). The intersecting genes were significantly enriched for chromatin remodelling related terms (Fig. 2d and Supplementary Table 4), which have previously been associated with ASD risk genes^8,24,73^. Overall, genes downregulated at 25 days in these four forms of ASD were enriched for known high-confidence ASD and NDD risk genes (16p11.2del: SFARI genes odds ratio (OR) 3.0, FDR = 3.8 × 10^−9^; 16p11.2dup: SFARI genes OR = 3.3, FDR = 1.4 × 10^−13^; PCDH19: SFARI genes OR = 3.1, FDR = 1.5 × 10^−5^; Timothy syndrome: SFARI genes OR = 2.0, FDR = 4.3 × 10^−2^; 16p11.2del: brain disorder genes OR = 2.4, FDR = 6.6 × 10^−3^; 16p11.2dup: brain disorder genes OR = 2.2, FDR = 1.2 × 10^−2^; PCDH19: brain disorder genes OR = 3.2, FDR = 2.2 × 10^−3^; Fisher’s exact test, Fig. 2e, Extended Data Fig. 6 and Methods). Gene set enrichment analysis (GSEA) found downregulation of terms related to neural precursor proliferation (for example, ‘Beta catenin binding’, ‘Neural precursor cell proliferation’ and ‘Canonical WNT signalling pathway’) in all 4 of these forms (Extended Data Fig. 6 and Supplementary Table 5). We detected only two significantly differentially expressed genes in the individuals with idiopathic ASD (*PRRC2C* at day 50 and the long non-coding RNA RP11-114H21.2 at day 100; FDR < 0.05).

Although the largest mutation-specific changes were observed early, the shared differential expression signature of mutational effects increased with maturation. Examination of the correlation of log_2_FCs over time revealed that the correlations between hCO harbouring different genetic events were significantly stronger at day 100 (analysis of variance (ANOVA) *F*_3,140_ = 11, *P* = 1.6 × 10^−6^; Tukey’s honestly significant difference (HSD): day 100 to day 25 *P* = 2.7 × 10^−5^, day 100 to day 50 *P* = 8.0 × 10^−6^, day 100 to day 75 *P* = 7.0 × 10^−4^; Fig. 2f,g) suggesting that the changes in gene expression converge as cortical differentiation progressed in vitro, with distinct mutational forms showing more consistent differences from control participants and less mutational specificity. Meta-analysis across forms found the highest number of significant genes at day 100, more than 50% greater than at day 25 (day 25, 4,034; day 50, 2,606 genes; day 75, 3,957 genes and day 100, 6,680 genes; Fig. 2h). This increase in convergence of gene-expression changes could not be explained by alterations in variability in control participants or the distinct genetic forms across development (Extended Data Fig. 4, *η*^2^ = 0.04).

## Dysregulated biological processes and cell types

**Fig. 3:**
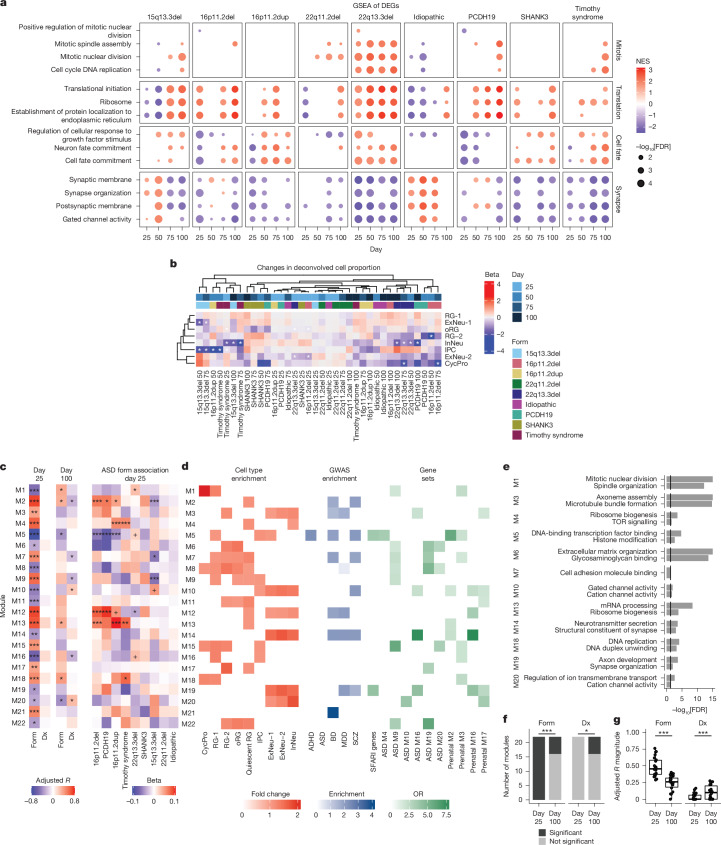
Pathways and cell types affected across forms of ASD. **a**, Select gene ontology terms enriched in upregulated (red) or downregulated (blue) genes in each ASD form. Colour shows normalized enrichment score (NES); point size shows −log_10_(FDR). Statistics were derived from GSEA. **b**, Cell proportion changes of ASD forms. Top annotation, differentiation day (shades of blue); lower annotation, ASD form (colourful). Statistics were derived from linear mixed-effects models and contrasts comparing each form with control participants with two-sided Wald tests. *FDR < 0.05. **c**, Adjusted *R* of WGCNA modules associated with either a form of ASD (Form) or with the presence of ASD (Dx) at day 25 and day 100 (left). Changes (linear model beta) in module eigengene in each ASD form (right). +FDR < 0.1, *FDR < 0.05, **FDR < 0.01, ***FDR < 0.005. **d**, Cell-type enrichment of modules using EWCE (red), genome-wide association study (GWAS) enrichment using a stratified linkage disequilibrium score (blue) and ASD-related gene set enrichment using a two-sided Fisher’s test (green). Only FDR < 0.05 are shown. **e**, Select gene ontology terms enriched in modules. Black lines represent FDR = 0.05. Statistics were derived from clusterProfiler enrichGO. **f**, The number of modules associated with either ASD forms (specific mutations) or with Dx. Significance was tested using two-sided Fisher’s exact test. **g**, Magnitude of module association with ASD forms or ASD broadly (Dx). Significance was tested using Welch’s two sample *t*-test (sample size: *n* = 22 modules per time point). Boxplots show: centre, median; lower hinge, 25% quantile; upper hinge, 75% quantile; lower whisker, smallest observation greater than or equal to lower hinge −1.5× interquartile range; upper whisker, largest observation less than or equal to upper hinge +1.5× interquartile range. ***P < 0.005. ADHD, attention-deficit/hyperactivity disorder; BD, bipolar disorder; CycPro, cycling progenitor; Dx, diagnosis; ExNeu-1, excitatory neurons 1 (early); ExNeu-2, excitatory neurons 2 (late); InNeu, interneurons; IPC, intermediate progenitor cells; MDD, major depressive disorder; ME, module eigengene; ORG, outer radial glia; RG-1, radial glia 1 (early); RG-2, radial glia 2 (late); SCZ, schizophrenia.

We observed that six different forms of ASD profiled were enriched for upregulated genes related to neuronal cell fate and protein translation-related terms by day 75 (ref.^ 74^) (Fig. 3a and Supplementary Table 5). We also observed broad downregulation of synapse and ion channel related terms such as ‘Synaptic membrane’ and ‘Gated channel activity’, across many time points and all the main mutational forms and in meta-analyses, particularly at day 75 and 100 (Fig. 3a, Extended Data Fig. 6 and Supplementary Table 5). The early disruption in pathways related to neurogenesis, neural progenitors and WNT signalling described above (for example, Fig. 2), coupled to the later emergence (day 75 and 100) of enrichment in synaptic pathways, provides a potential link between early mutational effects on neurogenesis that converge on signalling and synaptic biology during cortical neurogenesis across different genetic forms of ASD.

**Extended Data Fig. 7:**
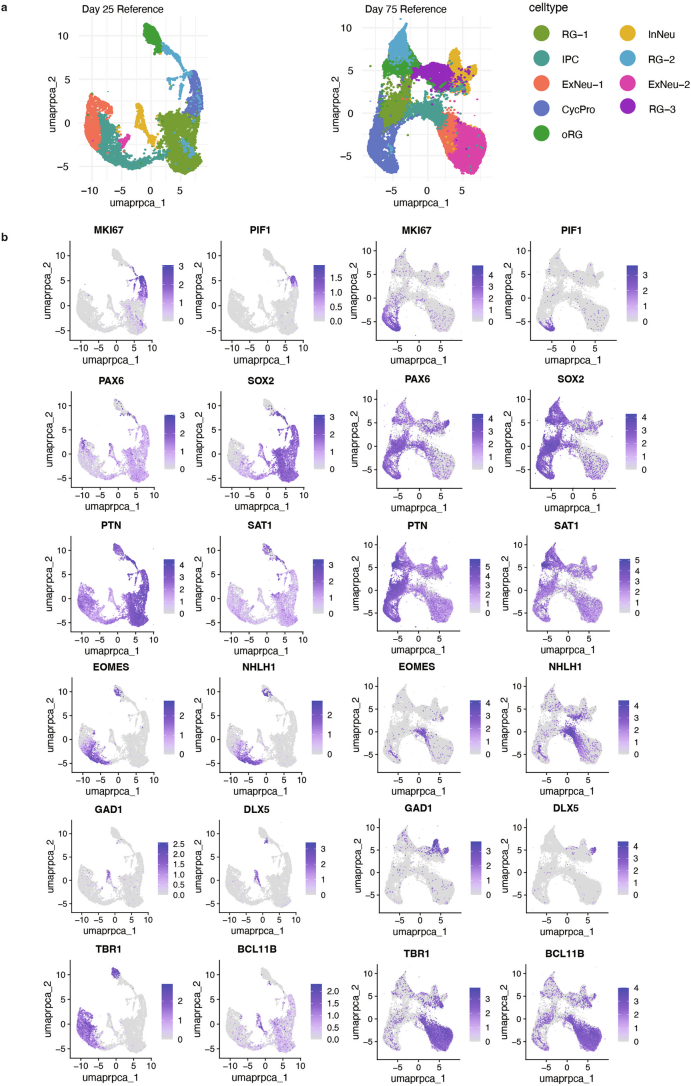
Single cell analysis of reference datasets for human cortical organoids. (**a**) Clustering of d25 organoid samples (left) and Clusters identified using samples from Khan et al. 2020^44^ (right). For Khan et al. samples, cluster characterization was based on control samples only. (**b**) Selected markers for Cycling progenitor (MKI67, PIF1; CycPro), Radial glia (PAX6, SOX2, PTN, SAT1; RG-1, RG-2, and RG-3), intermediate progenitors (EOMES, NHLH1; IPC), Interneurons (GAD1, DLX5; InNeu) and Excitatory neurons (TBR1, BCL11B; ExNeu-1 and ExNeu-2).

**Extended Data Fig. 8:**
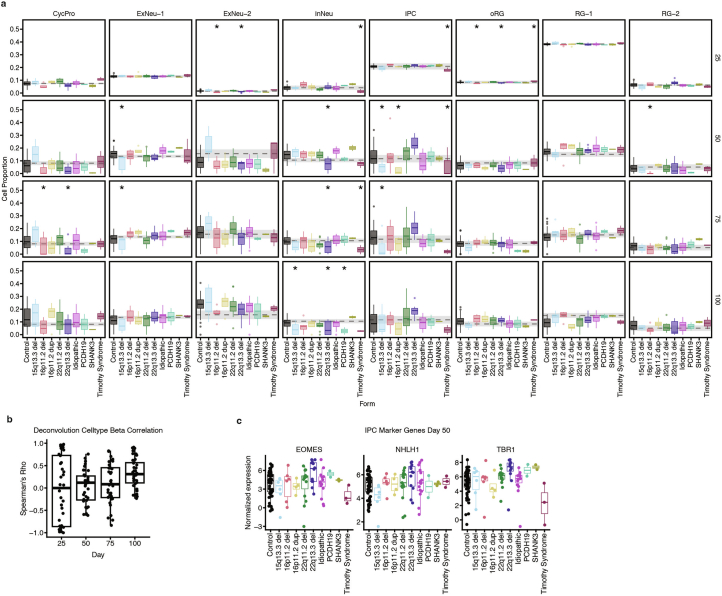
Deconvoluted cell proportions. (**a**) Cell proportion in each ASD form across hCO differentiation. Significant changes are marked by asterisk. CycPro - cycling progenitor, RG-1 - radial glia 1 (early), RG-2 - radial glia 2 (late), IPC - intermediate progenitor cells, ORG - outer radial glia, ExNeu-1 - excitatory neurons 1 (early), ExNeu-2 - excitatory neurons 2 (late), InNeu - interneurons. Grey bar indicates proportion of cell type in reference used to deconvolute. Stastics were derived from linear mixed-effects models and contrasts comparing each form to controls with two-sided Wald tests, *FDR < 0.05. Sample Numbers: Day 25: Controls – 46, 15q13.3del – 7, 16p11.2del – 5, 16p11.2dup – 4, 22q11.2del – 13, 22q13.3del – 11, Idiopathic – 12, PCDH19 – 2, SHANK3 – 2, Timothy Syndrome – 3. Day 50: Controls – 53, 15q13.3del – 6, 16p11.2del – 6, 16p11.2dup – 4, 22q11.2del – 15, 22q13.3del – 12, Idiopathic – 15, PCDH19 – 2, SHANK3 – 2, Timothy Syndrome – 3. Day 75: Controls – 54, 15q13.3del – 7, 16p11.2del – 6, 16p11.2dup – 4, 22q11.2del – 23, 22q13.3del – 12, Idiopathic – 15, PCDH19 – 2, SHANK3 – 2, Timothy Syndrome – 2. Day 100: Controls – 50, 15q13.3del – 6, 16p11.2del – 6, 16p11.2dup – 4, 22q11.2del – 15, 22q13.3del – 12, Idiopathic – 15, PCDH19 – 2, SHANK3 – 2, Timothy Syndrome – 2. (**b**) Correlation of deconvoluted celltype beta across time points. Day 100 cell type deconvolution effect is more correlated than day 25 (p-adj = 0.01). Stastics derived ANOVA followed by TukeyHSD post-hoc tests. (**c**) Normalized expression of IPC marker genes at day 50. Number of samples for each form: Control – 53, 15q13.3del – 6, 16p11.2del – 6, 16p11.2dup – 4, 22q11.2del – 15, 22q13.3del – 12, idiopathic – 15, PCDH19 – 2, SHANK3 – 2, Timothy Syndrome – 3. Boxplots a-c show: centre, median, lower hinge – 25% quantile, upper hinge – 75% quantile, lower whisker – smallest observation greater than or equal to lower hinge –1.5× interquartile range, upper whisker – largest observation less than or equal to upper hinge +1.5× interquartile range.

To further explore the relationships of gene-expression changes to specific cell types, we deconvolved bulk RNA-seq into cell-type transcriptomes using single-cell RNA-seq (scRNA-seq) from hCOs^44^ (Extended Data Fig. 7). Predicted changes in cell-type proportions were subtle and largely seen at one or two time points per form (Fig. 3b and Extended Data Fig. 8). The least mature progenitors (CycPros) and more mature neuronal cell types (ExNeu-2) showed predicted reductions in 16p11.2del and 22q13.3del, suggesting changes in the timing of neuronal maturation (ExNeu-2 decreased at day 25 and CycPro decreased at day 75), consistent with recent observations in a 16p11.2 organoid model^75^. Intermediate progenitors showed changes in 15q13.3 deletion, 16p11.2 duplication and Timothy syndrome, suggesting convergent effects on this cell type during development (Fig. 3b and Extended Data Fig. 8). Correlation of deconvoluted cell-type compositional shifts increased at day 100 compared with day 25, demonstrating convergence in cell-type changes, similar to what we observed with differential expression and changes in biological process (Extended Data Fig. 8). By day 100, although the correlation across forms increased, only InNeu showed any significant shifts from control participants in any given form (22q13.3del, 15q13.3del and PCDH19). This indicates that observed transcriptional convergence is probably related to both altered timing of differentiation of cell types and altered gene expression signatures within cell types, the latter appearing more prominent here.

**Extended Data Fig. 9:**
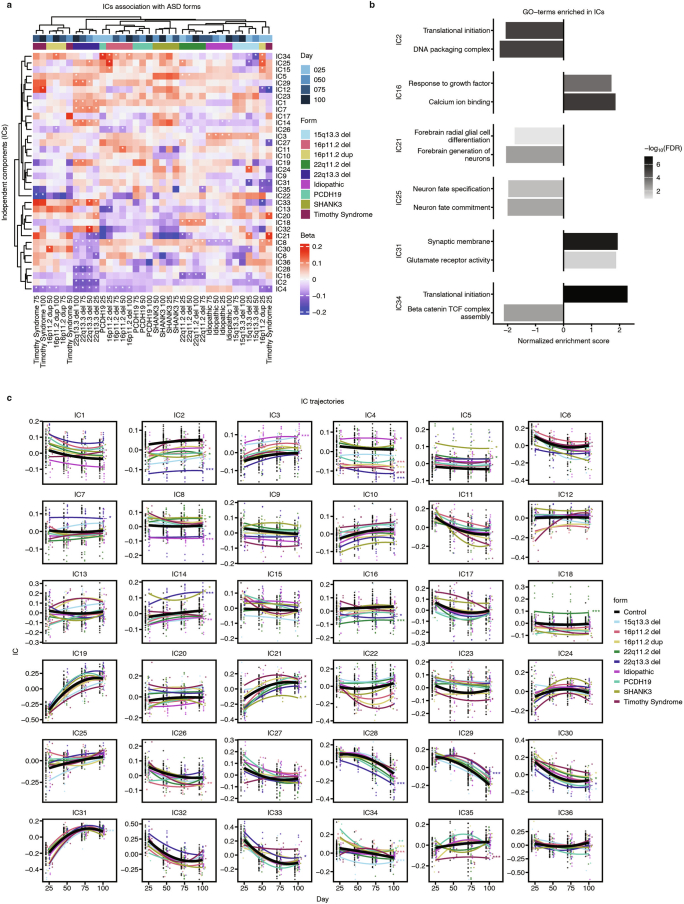
Independent Component Analysis. (**a**) Association of independent components (IC) with the different forms of ASD across time points. Stastics were derived from linear mixed-effects models and contrasts comparing each form to controls with two-sided Wald tests followed by FDR correction. *FDR < 0.05. (**b**) Select gene ontology (GO) terms enriched for the positively or negatively contributing genes for the indicated ICs. Negatively enriched terms are downregulated in ICs that are positively associated with forms of ASD and are upregulated in ICs that are negatively associated with forms of ASD. Stastistics were derived from GSEA. (**c**) Developmental expression of independent components (IC) for each ASD form. IC trajectory significance was tested using a linear model with polynomial splines with 2 degrees of freedom and 2 degrees of the piecewise polynomial. * FDR < 0.05, ** FDR < 0.01, *** FDR < 0.005.

We used an orthogonal method, independent components analysis to determine whether pathway changes were robustly identified; we observed high concordance between independent components analysis and expression-based clustering (Rand index 0.58, Extended Data Fig. 9). Upregulated independent components across 16p11.2 deletion and duplication were downregulated for processes involved in neuronal precursor renewal and differentiation, such as ‘Beta catenin-TCF complex assembly’ and ‘Neuron fate specification’^76^ (Extended Data Fig. 9 and Supplementary Table 6). The broad role of these pathways in ASD has previously been suggested^42,43,77^, highlighting their potential role as a point of convergence. Independent component 31 (IC31), enriched for synaptic terms, was downregulated in 16p11.2 duplication (IC31 beta = −0.13, FDR = 1.11 × 10^−3^) and Timothy syndrome (IC31 beta = −0.14, FDR = 2.9 × 10^−3^_;_ Extended Data Fig. 9 and Supplementary Table 6), similar to what was observed in the differential expression analysis.

## Network analysis supports convergence over time

**Extended Data Fig. 10:**
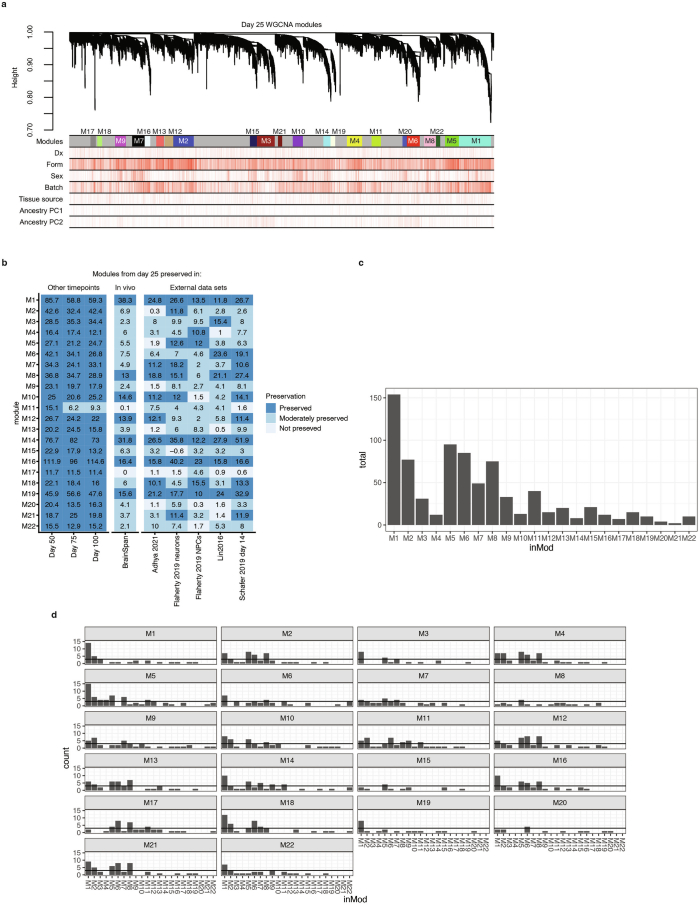
Construction and annotation of WGCNA modules. (**a**) Dendrogram from which the WGCNA modules at day 25 were derived using the dynamic tree cut algorithm^150^. Genes associations (adjusted R2) with covariates is also indicated. (**b**) Preservation of day 25 modules at other time points (left), in vivo (8-16 weeks post conception)^80^ and in other stem cell derived neuronal data sets: Adhya2020^81^, Flahrety2019^82^, Lin2016^83^ and Schafer 2019^84^. The Z_summary_ preservation score is indicated. (**c**) Number of TF-regulators (significantly enriched as regulators for at least 1 downstream module) within each module. (**d**) Number of TF-regulators within each module (x-axis) significantly regulating each downstream module (bar graph).

As transcripts do not act independently, but as part of highly regulated transcriptional networks^78^, we constructed co-expression networks using weighted gene co-expression network analysis (WGCNA)^78–80^. We tested whether modules derived from the day 25 samples changed in their association with individual forms and with all ASD-associated forms (diagnosis) compared with control participants over time (Fig. 3c, Extended Data Fig. 10 and Supplementary Table 7). Notably, we observed that the 22 identified co-expression modules from day 25 data were preserved at all other time points, suggesting they represent robust, continuing biological processes over this 100-day period. Moreover, their conservation in vivo^80^ and in vitro^81–84^ (Extended Data Fig. 10) further demonstrates that these modules represent generalizable human neurodevelopmental processes. At the earliest time point, there were 22 modules that differentiated between any of the forms of ASD and control participants. However, no single module differentiated diagnosis broadly from control participants (Fig. 3c and Supplementary Table 8). Eleven of the modules were significantly associated with at least one form of ASD, and five were associated with several (Fig. 3c–e). By contrast, at day 100, which corresponds to early mid-gestation^36,50,85^ and contains a large pool of postmitotic neurons and remaining radial glia, we identified only six modules that were associated with specific forms of ASD (Methods and Fig. 3c,f). However, at day 100, significantly more modules differentiated those participants who were affected (diagnosis) broadly from control participants (6 at day 100 compared with 0 at day 25; Fisher test *P* = 0.02; Fig. 3f) and the association of the modules with diagnosis broadly was significantly higher compared with day 25 (day 25 = 0.03, day 100 = 0.11, Welch two sample *t*_30.69_ = −3.0, *P* = 5.0 × 10^−3^; Fig. 3g), again demonstrating that the different genetic forms become more similar as differentiation progresses (Fig. 2f–h).

**Extended Data Fig. 11:**
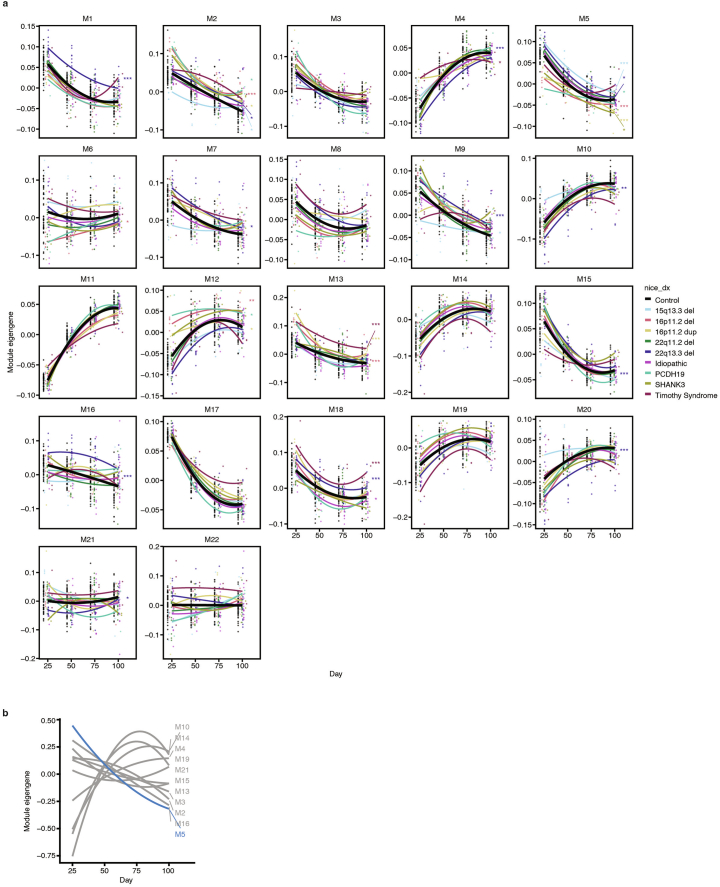
Module eigengene trajectories. (**a**) Module eigengene trajectories of day 25 modules across differentiation. Significance was tested using a linear model with polynomial splines with 2 degrees of freedom and 2 degrees of the piecewise polynomial. * FDR < 0.05, ** FDR < 0.01, *** FDR < 0.005. (**b**) Module eigengene trajectories of the regulators in M5 (blue) and the ASD risk genes that are regulated by these regulators (grey).

We found that two modules, M5 and M19, were significantly enriched for ASD risk genes (Fig. 3d M5: OR = 3.6, FDR = 9.4 × 10^−12^; M19: OR = 4.1, FDR = 2.1 × 10^−6^). This indicates that known ASD risk genes are strongly co-expressed within networks affected by perturbation of neural development in several rare forms of ASD, similar to observations based on in vivo data^17,24,25,29,86^. Most notable in this regard is M5, which was significantly downregulated across several forms of ASD at day 25 (adjusted *R* = −0.76, FDR = 5.2 × 10^−15^; 16p11.2 deletion beta = −0.06, FDR = 2.3 × 10^−6^; 16p11.2 duplication beta = −0.05, FDR = 5.6 × 10^−4^; PCDH19 beta = −0.08, FDR = 1.7 × 10^−5^) (Fig. 3c) and across differentiation (16p11.2 deletion: *t *= −3.5, FDR = 2.0 × 10^−3^; 16p11.2 duplication: *t* = −3.6, FDR = 2.0 × 10^−3^; PCDH19 *t* = −2.6, FDR = 2.0 × 10^−2^; SHANK3 *t* = −2.3, FDR = 3.8 × 10^−2^; Methods and Extended Data Fig. 11) suggesting that it has a central role downstream of these four ASD-associated mutations. M5’s relationship to in vivo human biology is supported by its significant overlap with two co-expression modules that peak during human fetal brain around mid-gestation that were previously identified as significantly enriched for rare ASD risk mutations^24^ (Fig. 3d: prenatal M2 OR = 5.8, FDR = 7.2 × 10^−32^; prenatal M3 OR = 2.0, FDR = 2.9 × 10^−4^). We observed that M5 was also enriched for genes co-expressed within a neuronal module that is downregulated in ASD postmortem cortex^14^ (Fig. 3d: ASD M4, OR = 3.5, FDR = 3.3 × 10^−5^), providing further evidence that it represents biological processes occurring in vivo in ASD. Functionally, M5 was enriched for terms related to regulation of gene expression such as ‘Histone modification’ and ‘DNA-binding transcription factor binding’ (Fig. 3e and Supplementary Table 8) and was enriched in developing cell types: early radial glia (RG-1; FC = 1.1, FDR = 4.5 × 10^−6^; Fig. 3d), intermediate progenitor cells (FC = 1.1, FDR = 4.5 × 10^−6^; Fig. 3d) and early cortical neurons (ExNeu-1; FC = 1.1, FDR = 3.0 × 10^−5^; Fig. 3d).

M19, the other module that was enriched for ASD risk genes, was downregulated across several ASD forms (day 25 adjusted *R* = −0.35, FDR = 0.01; Fig. 3c–e and Methods). M19 also overlapped with two later expressed, neuronal, human fetal cortical co-expression modules enriched for ASD risk^24^ (Prenatal M16 OR = 4.1, FDR = 1.7 × 10^−5^; Prenatal M17 OR = 2.7, FDR = 1.2 × 10^−3^; Fig. 3d). Functionally, M19 was enriched for terms related to synaptic function such as ‘Synapse organization’ and ‘Neurotransmitter secretion’, and neuronal cell types (ExNeu-1 FC = 1.5, FDR = 3.0 × 10^−6^; ExNeu-2 FC = 1.6, FDR = 3.0 × 10^−6^; InNeu FC = 1.5, FDR = 3.0 × 10^−6^; Fig. 3d,e).

## An upstream module regulating ASD risk genes

**Fig. 4:**
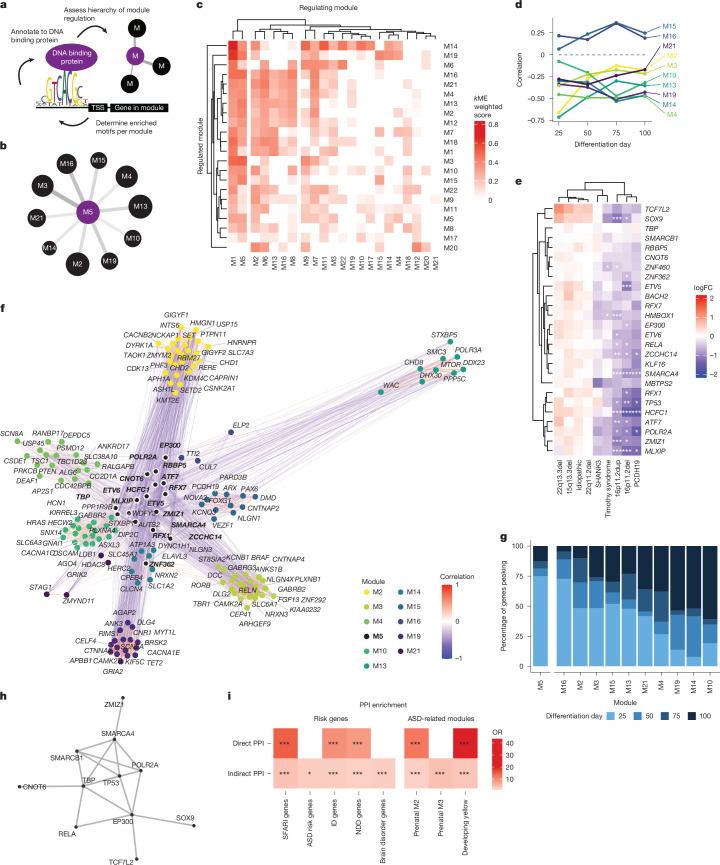
Module regulatory relationships reveal a key upstream regulator module. **a**, Workflow schema showing identification of enriched motifs (RcisTarget). Enriched motifs upstream of module genes are annotated to high-confidence DNA-binding genes. TSS, transcription start site. **b**, Modules predicted to be regulated by M5. Line width represents *k*ME weighted score, circle size represents proportion of genes regulated by M5. **c**, Heatmap of regulatory relationships between modules using a weighted *k*ME score to determine the strength of regulatory relationships between modules (*x* axis, regulating module; *y* axis, regulated module). **d**, Correlation of module 5 eigengene with downstream modules across differentiation, showing negative correlations with most downstream modules across time. **e**, Heatmap of logFC of gene regulators in M5 at day 25 shows strong decreases across several forms of ASD. Statistics are derived from dreamlet, * FDR < 0.05, ** FDR < 0.01, *** FDR < 0.005. **f**, Correlations of transcriptional regulators in M5 with ASD risk genes found in modules downstream of M5. M5 genes are in bold with a white ring. Most correlations are negative (blue) whereas genes within modules are positively correlated (red). **g**, Peak expression day of the regulators in M5 and the ASD genes that they regulate. Colour bars represent the percent of genes whose mean expression peak in the corresponding differentiation day. **h**, PPI of M5 regulator genes. These genes form a significant PPI network (*P* = 0.001, DAPPLE’s permutation test for the direct-edges network). **i**, Enrichment of regulators in M5 as well as their indirect network for ASD genes^2,8^ as well as NDD and intellectual disability risk genes^91^. Significance was tested using a two-sided Fisher’s exact test. *FDR < 0.05, **FDR < 0.01, ***FDR < 0.005.

As M5 met several criteria indicating it might be a factor in mediating convergence, including enrichment of early expressed ASD risk genes, significant differential expression across many forms, and enrichment for terms related to regulation of gene expression (Fig. 3c–e), we next tested the regulatory relationships between M5 and other modules associated with various forms of ASD (Fig. 4a and Methods). We determined the hierarchical regulatory relationships among modules (Fig. 4a, Supplementary Table 9, Methods and ref. ^87^), finding that M5 and M1 contained the highest levels of predicted upstream transcriptional regulators of other modules (Fig. 4b,c and Extended Data Fig. 10). Transcriptional targets of M5 were enriched in ten of the ASD-associated modules, among which was M19 (Figs. 3b and 4b). This highlighted M5’s potential role as a causal driver of the changes seen in the genetically defined forms of ASD, which is consistent with its expression at the earliest stages (Extended Data Fig. 11 and ref. ^24^).

Several modules downstream of M5 were associated with various processes known to be dysregulated in NDDs (Figs. 3d and 4b,c). We observed a relationship between M5 downregulation and the upregulated expression of genes in its downstream putative target modules, suggesting that M5 may be negatively regulating these modules (Fig. 4d). As genes in a module can vary in their degree of module membership^88^, we next asked whether the transcriptional regulators within M5 followed the same downregulation pattern. Indeed 65% (17 out of 26) of the annotated transcriptional regulators (Methods) in M5 were significantly downregulated in at least one of the forms of ASD with most (8 out of 9) of the other regulators trending in the same direction (Fig. 4e).

To further elaborate the relationship between M5 regulatory targets and ASD, we asked whether genes regulated by M5 were enriched for ASD genetic risk factors. Indeed, even though the individual co-expression modules containing these genes were not enriched for ASD risk genes, we found that the M5 regulated genes within M5 regulated modules were enriched for ASD risk genes (OR = 2.0, *P* = 3.6 × 10^−9^). A total of 261 SFARI 1, 2 or S genes within enriched downstream modules were predicted to be regulated by at least one M5 regulator (Fig. 4f and Methods). Noticeably, these ASD risk genes were mostly (68%) negatively correlated with the M5 regulators (Fig. 4f). Consistent with their role as transcriptional regulators, the driver genes within M5 peak in expression earlier than the ASD genes they are regulating (Fig. 4g). These M5 regulated ASD risk genes included many high-confidence risk genes associated with synapse-related function, such as *CNTNAP2* and *NLGN1* (M16), forebrain neuron differentiation, such as *FOXG1* and *PAX6* (M15), and epigenetic remodellers including *SET*, *DYRK1A*, *KMT2E* and *CHD2* (M2) (Fig. 4f), which are all processes previously related to ASD^2,24,89^. These genes also included *PCDH19* (M15) and *CACNA1C*, the gene mutated in Timothy syndrome (M10) (Fig. 4f), further reflecting this transcriptional network’s role as a point of convergence in ASD. Taken together, these findings suggest that a complex of transcriptional regulators orchestrates distinct biological pathways related to neurogenesis and neuronal maturation altered across genetically defined forms of ASD through regulatory relationships occurring during early stages of brain development.

## A regulatory network enriched for ASD risk genes

To determine whether these transcriptional regulators represent known biological processes, we asked whether they interact at the protein level. We found that they are predicted to form a highly significant protein–protein interaction (PPI) network (Methods; *P* = 0.001; Fig. 4h) enriched for transcription factor regulation and chromatin binding related gene ontology terms (Supplementary Table 10). Further examination of this PPI network indicates significant enrichment in known ASD and NDD risk genes (direct PPI, SFARI 1, 2, S genes OR = 15, FDR = 2.0 × 10^−4^; NDD OR = 8.4, FDR = 3.7 × 10^−4^; ID OR = 9.7, FDR = 2.0 × 10^−4^; Indirect PPI, SFARI genes OR = 3.7, FDR = 4.6 × 10^−11^; ASD risk genes OR = 2.4, FDR = 3.0 × 10^−2^; NDD genes OR = 2.1, FDR = 1.7 × 10^−7^; intellectual disability genes OR = 2.4, FDR = 1.9 × 10^−9^; brain disorder genes OR = 2.4, FDR = 3.6 × 10^−3^; Fig. 4i and Methods). Moreover, similar to the transcriptome-based M5, the PPI network strongly overlapped with the early to mid-gestation brain co-expression networks that have previously been associated with ASD^24^ (Direct PPI, Prenatal M2: OR = 12.3, FDR = 2.5 × 10^−4^; Fig. 4i), including common variant-enriched modules^29^ (Direct PPI, Developing Yellow: OR = 43, FDR = 7.1 × 10^−10^; Fig. 4i).

**Fig. 5:**
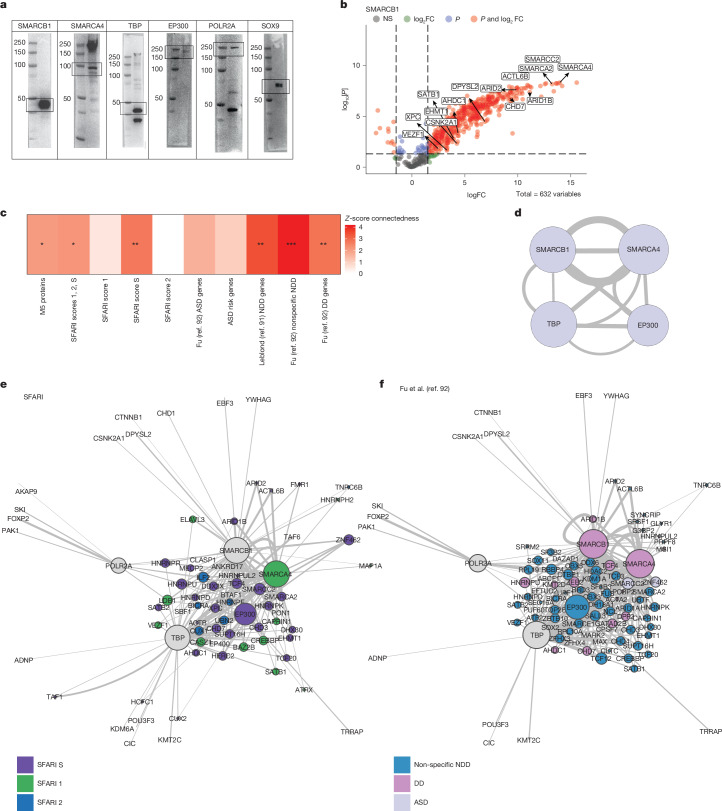
M5 regulators form a PPI network enriched for neurodevelopmental risk. **a**, Western blots (run once per antibody) confirm antibody binding for proteins of interest. **b**, Example volcano plot shows significantly bound proteins. Highlighted proteins are SFARI risk genes. Statistics were derived from three replicates using a two-tailed one-sample moderated *t*-test with FDR correction and a total of 632 variables. NS, not significant. **c**, *Z*-score connectedness of gene lists show that M5 proteins detected in IP–MS experiments form a significant PPI and that this network is significantly connected with other neurodevelopmental risk such as SFARI genes (1, 2, S), NDD genes^2,8,91^ and genes enriched for developmental delay (DD), ASD or NDD broadly^92^. *FDR < 0.05, **FDR < 0.01, ***FDR < 0.005, *z*-score computed against a permutation-derived null; FDR-corrected *P* values obtained from standard normal approximation. **d**, Experimentally determined PPI network of M5 proteins tested in IP–MS experiements. **e**–**f**, Experimentally determined PPI networks of the five M5 baits used showing their interactions with SFARI genes (**e**) and risk genes as defined in Fu et al.^92^ (**f**). Size of circle represents the number of connections, edge width represents logFC between nodes from one direction of the IP for each UniProt accession number. Colour represents the gene list. Grey nodes are network baits not in the featured gene list.

**Fig. 6:**
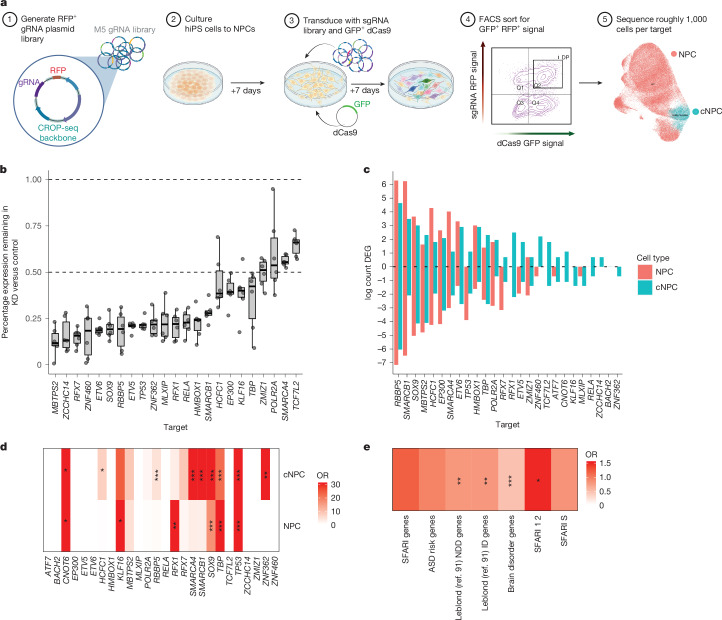
CRISPRi of M5 regulator genes shows effect on predicted targets and genes linked to NDDs. **a**, Schematic of experimental approach: 96 gRNAs corresponding to 26 genes and 18 non-targeting controls (NTCs) were designed and transduced into NPCs. Seven days posttransduction, single cells were sorted by fluorescence-activated cell sorting (FACS) to capture dCAS9–gRNA positive cells and scRNA-seq was performed (*n* = 6 replicates). NPCs were separated through single-cell clustering into cNPCs and NPCs. **b**, Ratio of target gene expression (*y* axis) remaining in target gRNA-expressing cells (*x* axis) compared to NTC. Boxplots show: centre, median; lower hinge, 25% quantile; upper hinge, 75% quantile; lower whisker, smallest observation greater than or equal to lower hinge −1.5× interquartile range; upper whisker, largest observation less than or equal to upper hinge +1.5× interquartile range. Dots show *n* = 6 replicate libraries. **c**, The log counts of DEGs (*P*_adjusted_  < 0.05, edgeR pseudobulk derived) in each target. **d**, Enrichment of predicted regulated genes in DEG dataset. **e**, Enrichment of DEGs in different groups of developmental disorder risk genes: SFARI genes (1, 2, S), ASD risk genes from refs. ^2,8^, NDD genes, intellectual disability risk genes and brain disorder genes from ref. ^91^. **d**,**e**, *FDR < 0.05, **FDR < 0.01, ***FDR < 0.005, Two-sided Fisher’s exact test. DP, double positive; GFP, green fluorescent protein; KD, knockdown; RFP, red fluorescent protein; sgRNA, single-guide RNA. Illustrations in **a** created in BioRender. Geschwind, D. (2025) (https://biorender.com/0pu4t7j).

**Extended Data Fig. 12:**
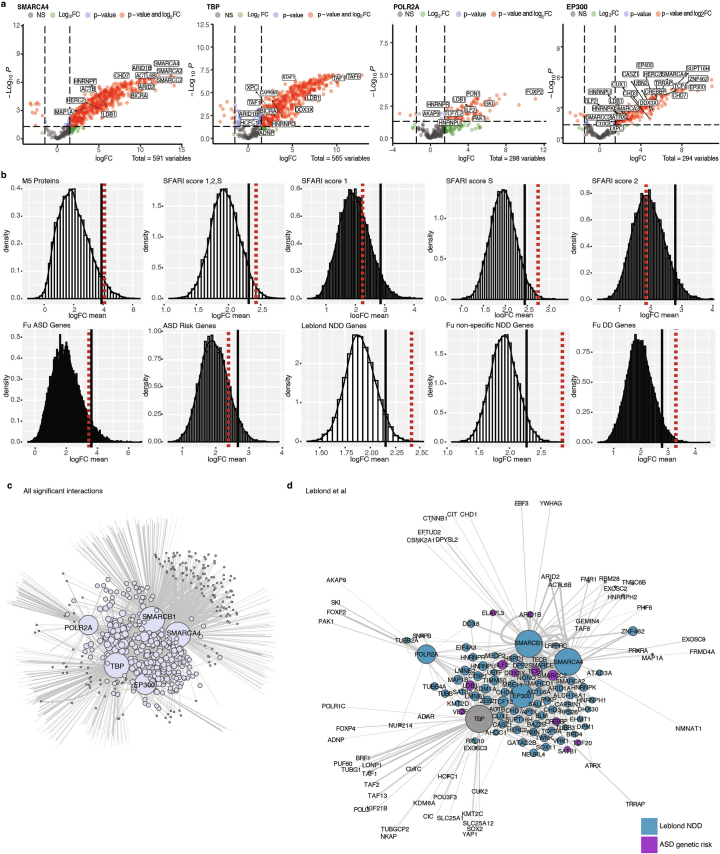
Immunoprecipitation followed by mass spectrometry (IP-MS) of M5 regulators. (**a**) Volcano plots show logFC of proteins compared to IGG controls. Highlighted are SFARI genes (1,2,S). Statistics were derived from 3 replicates using a two-tailed one-sample moderated *t*-test to calculate *P* values and FDR. (**b**) Significance of connectedness of networks is determined by comparing the network mean logFC to 10000 randomly selected networks of the same size across each of the protein baits. Red dotted line shows the network mean logFC and black line shows the 95^th^ percentile. (**c-d**) Connectivity networks from gene lists of interest. Size of circle represents number of significant proteins for each bait. **c)** Full connectivity of significant interactions of all baits. **d)** NDD and ASD risk genes^2,8,91^.

We next tested whether the predicted PPI interactions among M5 genes based on public databases (Fig. 4h–i) were observed in human neural progenitor cells (hNPCs), given that protein interactions can be highly cell-type specific and most available data are not from neural cell-types^90^. We conducted immunoprecipitation followed by mass spectrometry (IP–MS) on proteins with reliable antibody signal (Fig. 5a,b, Extended Data Fig. 12, Supplementary Table 11 and Methods). We then examined connections between M5 proteins (Methods), observing that M5 regulators do indeed form a significant PPI with each other and with a subset of the predicted interactors (Fig. 5c–f), including SFARI genes (score 1, 2 and S combined and score S; Fig. 5c,e), NDD genes^91^ (Fig. 5c,f and Extended Data Fig. 12) and genes enriched in developmental delay as well as NDD broadly^92^, validating database predictions (Fig. 4i). Of the five M5 regulators that passed quality control (Methods), we found that most were connected to three out of four of their possible connections, except for *POLR2A* (Fig. 6d). This PPI includes members of the BAF complex, which has a critical role during neurogenesis and mammalian cortical development^93–97^, *SMARCB1* (BAF47) and *SMARCA4* (BRG1), *EP300*, an important coactivator known to interact with BAF complex members^98^ and preinitiation complex member, *TBP*. These findings show that M5 regulators interact with a network of genes essential for early neurodevelopment, both in terms of predicted transcriptional control and direct PPI in neural progenitors.

## M5 regulators regulate their predicted targets

**Extended Data Fig. 13:**
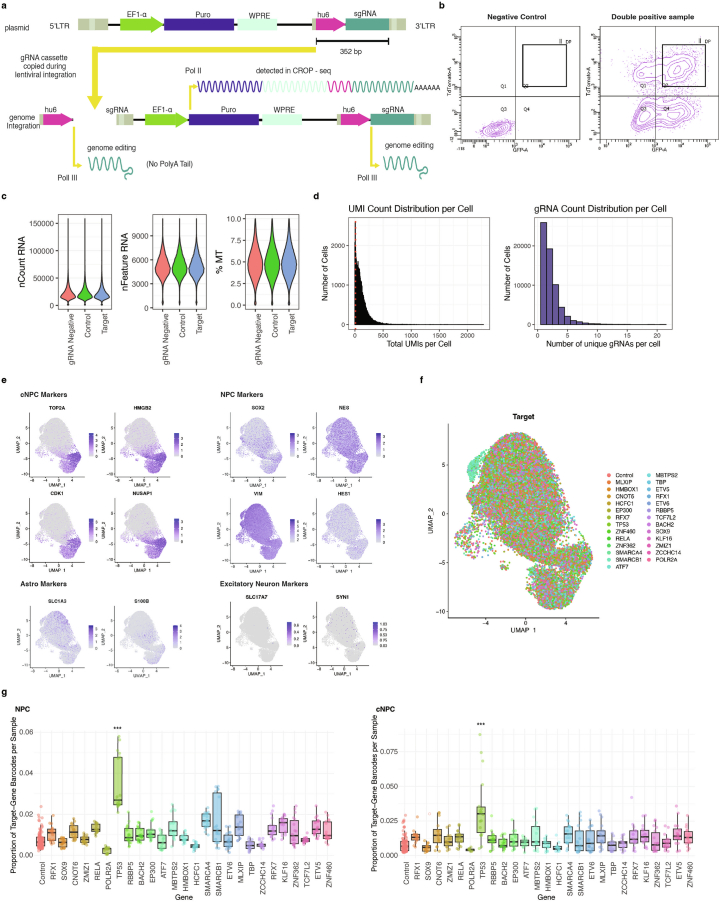
M5 Regulator CRISPRi methods and validation. (**a**) Schema of CROP-Seq vector. (**b**) Example of gating for flow cytometry. Left shows a set of hNPCs not expressing virus used for gating compared with hNPCs expressing both dCas9-Krab (GFP) and Crop-Seq-opti-dsRed (TdTomato). Box in the upper right shows double positive cells selected for downstream analysis (~20% of live cell fraction). (**c**) QC measures for CRISPRi single cell libraries organized by gRNA negative (no gRNA detected in sequencing), Control gRNA positive, or Target gRNA positive. (**d**) Number of gRNA UMIs per cell. X-axis shows cut-off of 10 UMIs used to consider a cell gRNA positive (left). Number of of unique gRNAs expressed in each cell (x-axis). Only cells expressing gRNA for a single gene-target are retained for downstream analyses. (**e**) Single cell UMAPs of markers used to differentiate cycling and non-cyling NPCs (**f**) Single cell UMAP coloured by Target-gene presence (**g**) Proportion of cells uniquely expressing gRNAs barcodes for each target within each experiment (n = 18 per Gene comprised of 6 technical replicates, 3 gRNAs barcodes per target, for Controls n = 108: 18 gRNAs across 6 technical replicates). TP53 show increased proportion compared to non-targeting controls (NTCs). Boxplots a-c show: centre, median, lower hinge – 25% quantile, upper hinge – 75% quantile, lower whisker – smallest observation greater than or equal to lower hinge –1.5× interquartile range, upper whisker – largest observation less than or equal to upper hinge +1.5× interquartile range. ***Family wise corrected p < 0.005, Dunnets post-hoc contrast of linear model.

Having validated components of the PPI network in developing neurons, we next sought to experimentally validate the regulatory role of M5 transcription factors and chromatin regulators on their predicted downstream targets. We leveraged the ability to perform a pooled, CRISPR–Cas9 inhibition (CRISPRi) screen to assess downstream gene expression on a single-cell basis^99^ (Methods, Fig. 6a and Extended Data Fig. 13). We applied this to neural cells derived by NGN2 induction in hiPS cells (hNPCs, Methods), targeting the promoters of 26 M5 putative transcriptional regulators using 3 guides per gene^100^ (Methods and Supplementary Tables 14 and 15). We achieved consistent downregulation (Fig. 6b and Extended Data Fig. 13), ranging from roughly 25% (*POLR2A*) to 90% decrease (*MBTPS2*) (Fig. 6b). hNPCs separated into mitotically active (NPCs showing consistent G2 mitosis markers (termed cycling NPCs or cNPCs), Extended Data Fig. 13), or those not expressing markers of active cell division (termed NPCs). For most targets, knockdown did not have a major impact on survival or proliferation, with 25 out of 26 knockdown targets showing no difference in proportion of cells compared to control gRNA-expressing cells, except for *TP53*, which showed increased proportions of cNPCs and NPCs (Extended Data Fig. 13), consistent with its well-known role as a tumour suppressor^101^.

**Extended Data Fig. 14:**
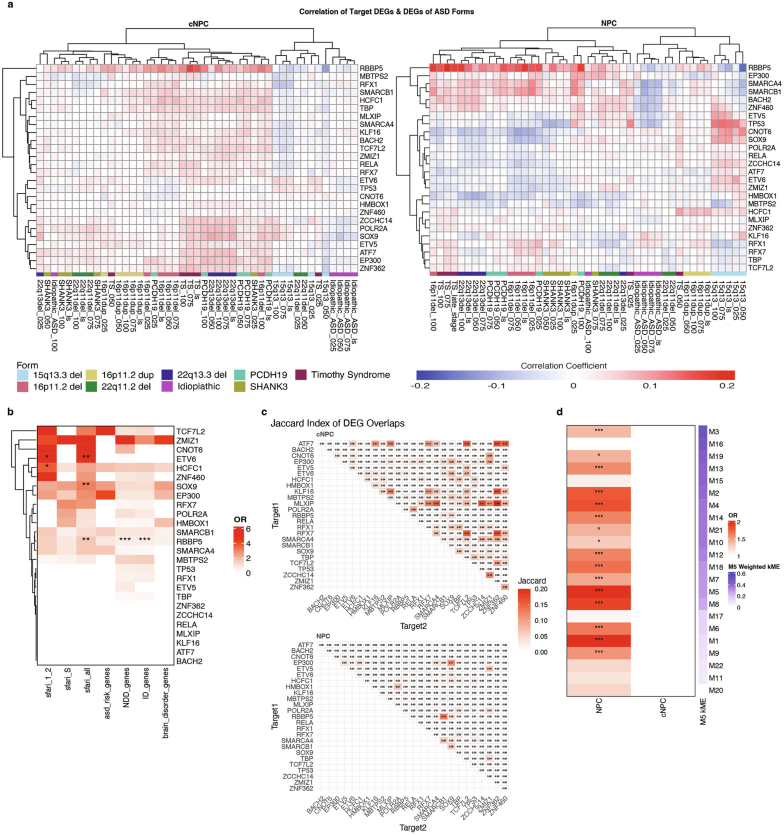
M5 Regulator CRISPRi additional data. (**a**) Broad positive correlation between DEGs across targets in NPCs (right) and cNPCs (left) and DEGs in patient-derived organoids across developmental stages. (**b**) Enrichment of rare genetic variation gene-lists in the differentially expressed genes of each target. Significance derived from two-sided Fishers exact tests. (**c**) Jaccard Index of DEG overlaps for each target. (**d**) Meta-analysis significant genes across targets show enrichment in several hCO-derived regulatory modules. Significance derived from two-sided Fishers exact tests.

**Extended Data Fig. 15:**
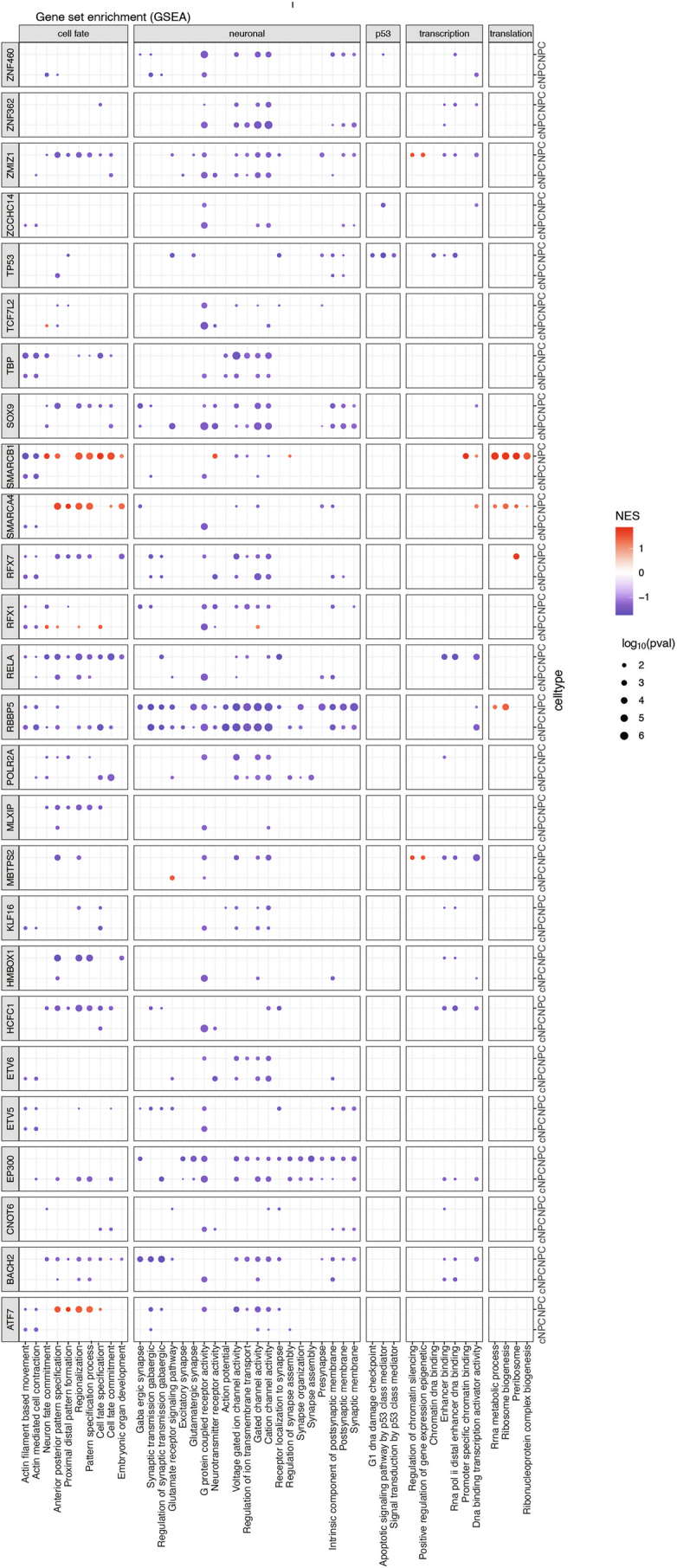
Gene ontology of CRISPRi targets. Gene Set Enrichment Analysis (GSEA) shows down-regulation of neuronal-related terms across M5 transcription factor regulators, particularly in RBBP5, SOX9, and EP300. Statstics were derived from fgsea R package.

We observed a wide spectrum of changes in gene expression following pooled CRISPRi, with *RBBP5* knockdown leading to more than 1,250 downregulated and more than 500 upregulated genes in NPCs to genes such as *RELA* and *BACH2* in which we observed no significant differential expression (Methods, Fig. 6c and Supplementary Table 12). *RBBP5* is part of the WRAD complex, which supports H3K4 methylation for SET-domain containing methyltransferases, many of which are NDD risk genes^102^. *RBBP5* knockdown led to massive changes in gene expression, including SWI–SNFI member *SMARCA1* and synapse genes *DLGAP1*, *NRXN3* and *CACNA1C*, the gene mutated in Timothy syndrome. Not every M5 regulator showed strong evidence for recapitulating phenotypes seen in hCOs, but knockdown of many M5 members produced a transcriptional response that was correlated with changes seen in organoids (Extended Data Fig. 14). Overall, knockdown of 11 of the 26 M5 regulatory drivers showed significant differential expression enrichment of their predicted targets: 9 out of 26 in cNPC and 6 out of 26 in NPCs, confirming their regulatory relationships (Methods, Fig. 6d and Extended Data Fig. 14). Parallel with the earlier bioinformatic predictions, we found that genes associated with NDDs and intellectual disability in addition to ASD risk genes^2,8,91^ (SFARI levels 1 and 2) were enriched in DEGs (Fig. 6e). Pathways downstream of several of the individual M5 regulators, including *RBBP5*,* EP300* and *SOX9* lead to disruptions of overlapping neurodevelopmental pathways (Extended Data Fig. 15 and Supplementary Table 13).

To determine the shared downstream effects of the M5 regulators, we performed a meta-analysis of single-cell differential expression from the pooled CRISPRi experiments. We observed that the genes significant in meta-analysis were enriched in several modules predicted to be regulated by M5 genes, including M2, M4 and M5 itself, suggesting regulation of other genes within the module by M5 regulators in NPCs (Extended Data Fig. 14). In summary, this examination of molecular responses to each M5 regulator perturbation, albeit in a single-cell type, was important to experimentally validate the bioinformatic predictions of regulatory relationships. These results indicate that further examination of these regulators in organoid models will help inform how changes in these genes alter cellular transitions during brain development.

We characterized the biological impact of several different genetic forms of ASD modelled in a large cohort of hCO, based on quantitative analyses rooted in genome-wide transcriptomic profiling. We identified robust clusters of different ASD forms spanning across stages of cortical development, suggesting several distinct developmental trajectories. Orthogonal analytic approaches showed that as time progressed these trajectories converged, consistent with canalization of development in which changes in the early stages of brain development are buffered as development progresses^103^. Overall, these trajectories converge on diminished neuronal maturation across time points, with most forms showing changing trajectories starting from day 25 and all, except for idiopathic ASD, showing changes by day 100. Although the alterations in maturation are convergent, the path to these changes seems to be distinct depending on different mutations, consistent with previous observations from a cohort of three other genetic forms of ASD^43^. Our findings suggest that convergence represents the impact of shared early developmental changes in co-expression networks enriched in ASD risk genes, whose downstream effects represent a form of developmental canalization on neuronal and synapse maturation. Our findings point towards known ASD risk genes associated with chromatin remodelling as a bridge linking early mutational specificity with convergence during cortical neurogenesis across different genetic forms of ASD.

We describe a core transcriptional network of driver genes acting at early stages and identified their downstream target biological processes. This core set of convergent genes is changed across several distinct genetic forms and is enriched in rare and common genetic risk for ASD. M5 dysregulation may not be limited to ASD, as it was also enriched for common variation associated with several psychiatric disorders^104,105^, and formed a PPI with neurodevelopmental risk genes broadly, reflecting the role of early neuronal differentiation in broad risk for psychiatric and NDDs. These gene regulatory relationships, which we show form interacting protein complexes, connect ASD risk genes across different pathways, including WNT signalling, translation and mTOR pathways, and synapse formation into a convergent network. It is also worth noting that we identified few consistent changes in lines from idiopathic ASD. This is probably due to the substantial polygenicity in ASD cases not harbouring major risk mutations^106,107^, and supports the need for much larger sample sizes in studies focused on idiopathic forms of ASD.

This work and other recent work^43,47^ demonstrates the utility of studying many mutations in parallel across developmental time to identify shared and distinct pathways in ASD. We note that the absence of effects in idiopathic ASD tempers our ability to identify broad, core ASD phenotypes that overlap across polygenic and major gene forms. Despite the comparatively large number of idiopathic lines profiled here, our data indicate that even larger samples will be needed to find robust shared phenotypes. However, the known overlap between genes associated with NDD and with ASD and our experimental strategy to examine several genetically defined forms of ASD in parallel, supports the relevance of these findings to ASD. By describing a core transcriptional network of driver genes, enriched in causal ASD genes and identifying their downstream targets, which are also enriched in ASD risk genes, these data connect ASD risk in genetically defined forms to convergent biological processes that occur during early cortical development.

## Methods

### Collection and characterization of hiPS cell lines

Control hiPS cell lines in this study were reported and have been extensively characterized using standardized methods^44,50,51^. hiPS cell lines from 11 idiopathic ASD individuals were obtained from Coriell. The 22q11.2 deletion^44^, 22q13.3 deletion^108^ and Timothy syndrome^45^ hiPS cell lines were reported and characterized before. To confirm the various genetic mutations, whole-genome sequencing was performed. Cultures were regularly tested for mycoplasma and maintained mycoplasma free. Approval for reprogramming and differentiation of these cell lines was obtained from the Stanford Institutional Review Board panel, and informed consent was obtained from all individuals.

### Reprogramming and hiPS cell culture

Human fibroblast cells were reprogrammed using the Sendai virus-based CytoTune iPS 2.0 kit (CytoTune 2.0, Invitrogen, A16517). Fibroblasts were plated in six-well plates 2 days before transduction to achieve a density of 2 × 10^5^ to 3 × 10^5^ cells per well. Cells were then transduced with the indicated set of Sendai vectors at a multiplicity of infection of 5–5–3 (*KOS*–*Myc*–*Klf4*) as we previously described in ref. ^44^. Virus was removed after 24 h and cells were cultured in fibroblast medium with 10% FBS (Gibco, 16000-044) in Dulbecco’s modified Eagle medium (DMEM) (Gibco, 10569-010) for a total of 7 days, with media changes every other day. On day 7 after transduction, cells were collected with TrypLE (Gibco, 16563-029) and plated in the fibroblast medium onto truncated recombinant human vitronectin (VTN-N, Gibco, A14700)-coated plates. The following day, fibroblast medium was changed to Essential 8 medium (Gibco, A1517001) and cells were fed daily until days 20–22. For further culture, individual colonies were manually dissected and transferred to fresh vitronectin-coated dishes. Once hiPS cell clones were established, they were routinely cultured on vitronectin-coated dishes in Essential 8 medium, and cells were split every 4–5 days using 0.5 mM EDTA (Invitrogen, 15575-020). hiPS cell clones and lines were also generated using retroviruses or non-integrating episomal vectors from fibroblasts^109^. We did not observe any systematic differences between cells reprogrammed by the two methods. hiPS cells were cultured as previously described in ref. ^51^. Briefly, hiPS cells were cultured on six-well plates coated with recombinant human vitronectin (VTN-N, Gibco, A14700) in Essential 8 medium (Gibco, A1517001) and passaged with 0.5 mM EDTA (Gibco, 15575). To coat the six-well plates, 1 ml of vitronectin (diluted at a 1:100 ratio with Dulbecco’s phosphate-buffered saline (DPBS); Gibco, 14190) was added per well and then incubated at room temperature for 1 h. To passage hiPS cells with 80–90% confluency, cells were rinsed with 3–4 ml of DPBS per well, and then 1 ml of 0.5 mM EDTA (Gibco, 15575) was added and incubated for 7 min at room temperature. After the EDTA was removed, 2 ml of prewarmed complete Essential 8 medium was added to collect cells. The cell suspension was then diluted in Essential 8 medium (1:6–1:20 depending on the hiPS cell line) and distributed on vitronectin-coated wells.

### Generation of hCOs from hiPS cells

For the generation of 3D spheroids, hiPS cells were incubated with Accutase (Innovate Cell Technologies, AT-104) at 37 °C for 7 min and dissociated into single cells. To obtain uniformly sized spheroids, we used AggreWell 800 plates (STEMCELL Technologies, 34811) containing 300 microwells. There were roughly 3 × 10^6^ single cells per well in Essential 8 medium supplemented with the ROCK inhibitor Y-27632 (10 μM, Selleckchem, S1049), centrifuged at 100*g* for 3 min to capture the cells in the microwells and incubated at 37 °C with 5% CO_2_. After 24 h, day 0 of differentiation, spheroids were collected from each microwell by firmly pipetting (with a cut end of a P1000 tip) medium in the well up and down, and transferring it into ultra-low-attachment plastic dishes (Corning, 3262) in Essential 6 medium (Gibco, A1516401) supplemented with two SMAD pathway inhibitors: dorsomorphin (2.5 μM, Sigma-Aldrich, P5499) and SB431542 (10 μM, Tocris, 1614) together with Wnt pathway inhibitor XAV939 (1.25 μM, Tocris, 3748). Media changes were performed daily, except for day 1. On the sixth day in suspension, neural spheroids were transferred to neural medium containing Neurobasal-A (Gibco, 10888), B-27 supplement without vitamin A (Gibco, 12587), GlutaMax (1:100, Gibco, 35050) and penicillin and streptomycin (1:100, Gibco, 15140). The neural medium was supplemented with epidermal growth factor (20 ng ml^−1^, R&D Systems, 236-EG) and basic fibroblast growth factor (20 ng ml^−1^, R&D Systems, 233-FB) until day 24. From days 25 to 42, to promote differentiation of the neural progenitors into neurons, the neural medium was supplemented with brain-derived neurotrophic factor (20 ng ml^−1^, Peprotech, 450-02) and neurotrophin 3 (20 ng ml^−1^, Peprotech, 450-03), with medium changes every other day. From day 43 onwards, only neural medium without growth factors was used for medium changes every 4–5 days.

### RNA-seq processing

Sequencing libraries were prepared using TruSeq stranded RNA RiboZero Gold (Illumina) on ribosomal RNA-depleted (RiboZero Gold, Illumina) RNA. The libraries were sequenced with 100-base-pair paired-end reads on an Illumina HiSeq 4000. The reads were mapped to the human genome (hg38) with Gencode v.25 annotations using STAR (v.2.5.2b)^110^ and gene expression was quantified using RSEM (v.1.3.0)^111^. Genes that were expressed at very low levels (fewer than 10 reads in 30% of samples from a given day) were removed from the analysis. Samples with standardized sample network connectivity *Z* scores below −2 in each mutation were defined as outliers and removed^112^. To control for technical variation due to the sequencing and library prep we calculated the principal components of the Picard sequencing metrics (http://broadinstitute.github.io/picard/) using the CollectAlignmentSummaryMetrics, CollectRnaSeqMetrics and MarkDuplicates modules, and included them in our model. To infer genetic ancestry, we called single-nucleotide polymorphisms (SNPs) from the aligned reads using the GATK (v.3.3) Haplotype caller^113^. Sites with more than 5% missing samples, with rare minor allele frequency (less than 0.05) and Hardy–Weinberg disequilibrium (less than 1 × 10^−6^) were removed using plink (v.1.09)^114^. We then used the remaining high-quality SNPs to run MDS together with HapMap3.3 (hg38)^115^. The first two MDS values, referred to as genetic ancestry principal components 1 and 2 (PC1, PC2), were then included in our model. Sample identity was verified using the identity by descent algorithm from PLINK (v.1.09)^114^. Identity by descent was calculated for each pair of samples based on genotypes derived from RNA-seq analysis as well as for all pairs of RNA-seq and DNA sequencing samples. Samples with a $\hat{\pi }$ < 0.8 from other samples derived from the same individual were removed. Sex was verified for each sample by detection of genes expressed by the Y chromosome.

Sample with high levels of duplication (Picard tools, PERCENT_DUPLICATION > 65), high levels of intergenic mapping (Picard tools, PCT_INTERGENIC_BASES > 60) or with low levels of messenger RNA (RNA) (Picard tools, PCT_MRNA_BASES < 0.5) were removed. All RNA-seq samples passing quality control are listed in Supplementary Table 1. Variance partitioning was performed using the variancePartition package (v.1.20.0) with default parameters. Gene-expression reproducibility was measured using Spearman’s correlation between every pair of samples in a given time point and mutation both within and between individuals.

### Whole-genome sequencing

Libraries were prepared using TruSeq DNA PCR-Free (Illumina) and were sequenced with 150-base-pair paired-end reads on an Illumina HiSeq 4000. Lines that underwent whole-genome sequencing are in Supplementary Table 2: further lines were quality controlled previously^44^. Sequencing data were processed as previously described in ref. ^8^.

The sequencing reads were mapped to the human genome (hg38) using Burrows–Wheeler Aligner (bwa-mem, v.0.7.17)^116^. The resulting BAM files were merged using bamtools (v.2.5.1)^117^ resulting in a single BAM file per sample. Duplicate reads were marked using the Picard MarkDuplicates tool (v.2.5.0). Reads around indels were locally realigned using GATK’s IndelRealigner (v.3.3). Base quality score was recalibrated using GATK^113^. Variant and non-variant bases in the genome were identified using GATK’s HaplotypeCaller (v.3.3). Resulting variant cal formats were combined using GATK’s combineGVCFs (v.3.3) and variants were called per chromosome using GATK’s GenotypeGVCFs (v.3.3). Well-calibrated quality scores were generated using GATK’s Variant Quality Score Recalibration (v.3.3). Large chromosomal changes, resulting from iPS cell chromosomal instability, were identified using DELLY (v.0.8.7)^118^. Lines with large indels (more than 750 kb) that were confirmed using allele frequency in the RNA-seq data were removed from the analysis. This included five individuals in whom all lines were dropped (four because of incorrect or absent mutations, one that did not pass quality control). Twenty-six lines in total (19 individuals) were dropped due to quality control failures. These include seven lines that were dropped due to failure to confirm mutation (four individuals), nine lines dropped due to large CNVs (seven individuals), two lines dropped due to leftover Sendai virus (two individuals), four due to further or improper deletions (four individuals) and four that were not fully characterized or did not pass quality control (two individuals). Point mutations in *SHANK3*, *PCDH19* and *CACNA1C* were visually confirmed using Integrative Genomics Viewer (v.2.9.4)^119^. Sequencing depth was calculated using Samtools depth command (v.1.9)^120^. All whole-genome sequencing samples are listed in Supplementary Table 2.

### Differential expression

Gene counts were normalized using trimmed mean of *M* values^121^ as implemented in the calcNormFactors function from the edgeR package (v.3.26.8)^122^. The mixed linear model used included differentiation day, sex, batch, the first 2 principal components calculated for genetic ancestry and 15 sequencing principal components (SeqPCs) as fixed effects, and accounted for many samples coming from the same individual, hiPS cell line and differentiation, and for samples that were sequenced several times as random effects. The model was implemented using the dream function from the variancePartition package (v.1.20.0)^123^. Genes with FDR < 0.05 were considered to be differentially expressed. To conduct meta-analyses on mutational differential gene expression, we used the metafor (v.4.6.0) package by fitting a fixed-effect model with the rma function for each time point^124^. FDR < 0.05 genes were considered to be differentially expressed. GSEA was run on metafor analysis output to determine enriched pathways using the fgsea package (v.1.3.0).

### Independent component analysis

Independent component analysis was performed using the JADE algorithm as implemented in the MineICA package (v.1.30.0)^125^. The number of components was set to 36 with 10,000 maximum iterations. Association of the independent component with the forms of ASD was tested using a mixed linear model. The model was implemented using the lme function from the nlme package (v.3.1.152). Contrasts were used to compare each form of ASD at each time point to the control at the same time point using the glht function from the multcomp package (v.1.4.18). Like other component analyses, independent component directions are random and so enrichment was performed for both positively and negatively contributing genes. Unlike principal components, independent components are not inherently ordered and were therefore ordered by the amount of kurtosis in each independent component.

### GSEA

GSEA was performed using the fgsea package (v.1.10.1)^126^ on all genes with 1,000,000 permutations and a set size ranging from 30 to 500. Gene ontology gene sets (v.7.0) were downloaded from http://software.broadinstitute.org/gsea/msigdb/. For differential expression genes were ranked by log_2_FC and for independent component analysed genes were ranked by the projection values of the genes on each component. Enrichment for high-confidence ASD genes (gene score of two or less, or syndromic genes) from the SFARI database (https://gene.sfari.org/database/gene-scoring/), from large scale ASD sequencing studies^2,8^, and risk genes for NDDs, intellectual disability and brain disorders^91^ was performed using Fisher’s exact test.

### Network analysis

Gene counts were normalized using conditional quantile normalization (v.1.36.0)^127^ and the covariates sex, tissue of origin, genetic ancestry PC1 and PC2 and the first 15 technical sequencing principal components were regressed out. The network was constructed using the WGCNA package (v.1.70.3)^88^ using a signed network for each time point separately. A modified version of robust WGCNA was used to reduce the influence of potential outlier samples on network architecture^26^. Samples were resampled from within each form of ASD ensuring that each iteration contained at least four samples from each form. In total, 100 networks were constructed followed by consensus network analysis that takes the median of the topological overlap dissimilarity matrix across the 100 networks^88^. Soft-threshold powers of 13, 9, 13 and 14 were used for days 25, 50, 75 and 100, respectively, to achieve approximate scale-free topology (*R*^2^ > 0.8). Modules were constructed using a minimal module size of 160, deep split of 4, cut height for creation of modules of 0.9999 and cut height for merging modules of 0.1. Each module eigengene (first principal component of the module) was tested for association with the different forms of ASD as well as for association with ASD overall using linear models. Module gene-ontology term enrichment was performed using clusterProfiler (v.4.0.5)^128^ with default parameters. Enrichment for common variation associated with ASD^21^, schizophrenia^129^, attention-deficit/hyperactivity disorder^130^, major depressive disorder^131^ and bipolar disorder^132^ was calculated using a stratified LDscore regression (v.1.0.0)^133^. SNPs with 10 kb of a gene were assigned to that gene and the enrichment was calculated as the proportion of SNP heritability accounted for by the genes in the module divided by the proportion of total SNPs within the module. Cell-type enrichment was performed using the bootstrap enrichment method from the EWCE package (v.1.0.1)^134^ using control samples from previously published single-cell data from a human cortical spheroids set^44^ with 100,000 permutations. As the background gene set, all genes expressed in both the current dataset and the single-cell dataset were used. Enrichment for high-confidence ASD genes (gene score of two or less or syndromic genes) from the SFARI database (https://gene.sfari.org/database/gene-scoring/) and ASD postmortem modules^14^ was performed using Fisher’s exact test. Module trajectories were modelled using polynomial splines with two degrees of freedom and two degrees of the piecewise polynomial and tested for significant differences using a linear model. Module preservation was performed using the modulePreservation function from the WGCNA package with 100 permutations. Preservation was tested (1) at other time points within the current data set (2) in cortical samples from early developmental stages (8–16 weeks postconception) from the BrainSpan in vivo data set and (3) in other stem cell-based neuronal models^81–84^. To test the association of each ASD form with WGCNA modules, we ran a linear regression comparing the module eigengene of each module by form (as factor variable with control participants as the base level) or by diagnosis (affected versus control) using the lm() function. Adjusted *R* values of the regression are multiplied by the sign of the sum of model coefficients to determine direction of effect. Estimates for individual forms were derived from the model coefficients. We show FDR-corrected *P* values in the figures.

### Clustering stability

The different conditions (ASD form/differentiation day) were clustered based on the Spearman correlation of log_2_FC of all expressed genes using the Ward.D2 clustering method on Euclidean distances as implemented in the ComplexHeatmap package (v.2.9.3)^135^. Cluster significance was tested using multiscale bootstrap resampling as implemented in the pvclust function in the pvclust package (v.2.2.0)^136^ using the Ward.D2 clustering method on Euclidean distances with 10,000 bootstraps. The bootstrap probability measure is the frequency that a cluster appears in the bootstrap replicates. To test for clustering similarity across clustering methods, clustering was performed using the Ward.D, Ward.D2, complete, average and Mcquitty methods on each of the Euclidean, maximum, Manhattan and Minkowski distances. For each pair of clustering and distance methods the cophenetic correlation was measured using the cor.dendlist function from the dendextend package (v.1.14.0)^137^. Cophenetic correlation is a measure of how well the pair-wise distances of the unmodeled data are preserved in the dendrogram^138^. In addition, the Rand index was measured for each combination of these pairs using the rand.index function from the fossil package (v.0.4.0)^139^. Rand index is a measure of the similarity between two trials of clustering using the same data^140^. To verify that the clusters were not driven by a small subset of genes, we subset the genes used for clustering to 10,000, 5,000 or 2,000 random genes 100 times. The resulting genes were correlated using Spearman correlation and clustered using the Ward.D2 clustering method on the basis of Euclidean distances. Cophenetic correlation and Rand indexes were calculated as above.

### Deconvolution

Bulk RNA-seq counts were deconvolved using the control cell profiles from previously published single-cell data^44^ from day 75–85 organoids to deconvolve day 50, 75 and 100 time points, and new single-cell libraries were created with standard 10X methods from day 25 organoids to deconvolve the day 25 time point. Single-cell datasets were processed and integrated separately using standard Seurat v.5 workflows. The reference-based decomposition was performed using the ReferenceBasedDecomposition function from the BisqueRNA package (v.1.0.5)^141^. Changes in cell-type proportion were tested using a logit mixed linear model. The logit transformation was performed using the logit function from the car package (v.3.0.11) with a 0.001 adjustment. The model was implemented using the lme function from the nlme package (v.3.1.152). Contrasts were used to compare each form of ASD at each time point to the control at the same time point using the glht function from the multicomp package (v.1.4.18).

### Transcription factor binding over-representation

To identify transcription factor binding motifs over-represented in each module we used RcisTarget (v.1.6.0)^142^. The transcription factor data base used was hg38 and included 10 kb upstream and downstream of the transcription start site (version 9; hg38__refseq-r80__10kb_up_and_down_tss.mc9nr). Only high-confidence annotated transcription factors were used for downstream analysis. The regulatory relationship between modules was calculated as a *k*ME (which is the correlation of each gene in the module with the module eigengene) weighted proportion of the number of genes regulated by the upstream module in the downstream module such that: $\mathrm{score}=\frac{\sum k\mathrm{ME}\,\mathrm{of}\,\mathrm{regulated}\,\mathrm{genes}\,\mathrm{in}\,\mathrm{the}\,\mathrm{module}}{\mathrm{Total}\,\mathrm{number}\,\mathrm{of}\,\mathrm{genes}\,\mathrm{in}\,\mathrm{the}\,\mathrm{module}}$. This weighted proportion puts larger weight on genes that are highly connected within the module. A module was considered to be highly regulated by another module if the phosphastaseME weighted score was above 1 standard deviation above the mean (which corresponded to a weighted score of 0.341).

### PPIs

PPIs were mapped using the Dapple^143^ module (v.0.19) in GenePattern (http://genepattern.broadinstitute.org) using the hg19 genome build with 1,000 permutations.

### IP–MS

To validate predicted PPIs, we generated mass spectrometry data from key regulator proteins predicted to form a PPI. We tested antibodies against RELA (Active motif 40916), TP53 (Abcam ab26), CNOT6 (CST no. 13415), TBP (CST no. 8515), POLR2A (PTG 20655-1-AP), SOX9 (CST no. 82630), SMARCB1 (CST no. 91735), SMARCA4 (Abcam ab110641) and EP300 (Abcam ab275378). All antibodies were run at 1:1,000 concentrations. Antibodies that generated western blot bands at the appropriate size were selected for continued mass spectrometry analysis (run once; Fig. 5a). For western blot analysis of the proteins of interest, cell lysates were diluted in 6× SMASH buffer (5 mM Tis HCL pH 6.8, 10% glycerol, 2% SDS, 0.02% bromophenol blue and 1% beta-mercaptoethanol) and boiled for 10 min at 95 °C. Samples were then separated on a NuPAGE 4–12% Bis-Tris Protein Gel (Invitrogen) and transferred to a polyvinyl difluoride membrane (Life Technologies, 100 V, 2 h). Membranes were incubated at room temperature for 1 h in tris-buffered saline with Tween (TBST) (0.1% Tween) with 5% BioRad Blotting-grade Blocker and then incubated overnight with antibodies listed above at 4 °C. After 3× 10-min washes in TBST, blots were incubated with horseradish peroxidase (HRP)-conjugated secondary antibodies for 45 min and again washed three times in TBST. The following secondary antibodies were used: anti-mouse IgG HRP-linked (NA9310V, Sigma-Aldrich) and anti-rabbit IgG HRP-linked (GENA934, Sigma-Aldrich) at a concentration of 1:5,000. Western blots were imaged using SuperSignal West Femto Maximum Sensitivity Substrate (Thermo Scientific). Western blots were considered successful if the band was visualized at the expected molecular weight (Fig. 5a). For the 6 proteins that showed successful western blots, 1–2 mg of protein extract was incubated with primary antibody (1–2 µg) at 4 °C overnight (1 µg of primary antibody to 1 mg of protein). Following primary antibody incubation 100 µl of Protein A/G beads (Pierce) were added and incubated for 4 h at 4 °C, followed by 1 wash with lysis buffer (Pierce) with added Halt protease and phosphatase inhibitors (Thermo Scientific), 2 washes with PBS and then resuspended in PBS for mass spectrometry.

### Mass spectrometry and quantification

Samples were processed and quantified for mass spectrometry as in ref. ^90^ for the 6 proteins (TBP, POLR2A, SOX9, SMARCB1, SMARCA4 and EP300) with a western blot signal in NGN2-derived NPCs at day 4 (refs. ^90,144,145^). Briefly, NPCs were collected at day 4 following NGN2 expression and dual SMAD/WNT inhibition in the presence of doxycycline (rtTA, tetON-NGN2). Cells were pelleted, washed with PBS and resuspended in 10× packed cell volume immunoprecipitation lysis buffer (Thermo Scientific) with added Halt protease and phosphatase inhibitors (Thermo Scientific) for 20 min at 4 °C. Cells were then centrifuged (16,200*g*, 20 min, 4 °C and resuspended in 3× packed cell volume. Samples were quantified using the Thermo BCA protein assay. Immunoprecipitations were eluted using SDS and subject to electrophoresis for gel extraction. Bands were excised and cut into roughly 1 mm^3^ pieces. Gel pieces were then subjected to a modified in-gel trypsin digestion procedure, washed and dehydrated with acetonitrile for 10 min, followed by removal of acetonitrile. Pieces were then completely dried in a speed-vac. Rehydration of the gel pieces was with 50 mM ammonium bicarbonate solution containing 12.5 ng µl^−1^ modified sequencing-grade trypsin (Promega) at 4 °C. After 45 min, the excess trypsin solution was removed and replaced with 50 mM ammonium bicarbonate solution to just cover the gel pieces. Samples were then placed in a 37 °C room overnight. Peptides were later extracted by removing the ammonium bicarbonate solution, followed by 1 wash with a solution containing 50% acetonitrile and 1% formic acid. The extracts were then dried in a speed-vacuum (roughly 1 h). The samples were then stored at 4 °C until analysis.

On the day of analysis, the samples were reconstituted in 5–10 µl of high-performance liquid chromatography solvent A (2.5% acetonitrile, 0.1% formic acid). A nano-scale reverse-phase high-performance liquid chromatography capillary column was created by packing 2.6-µm C18 spherical silica beads into a fused silica capillary (100 µm inner diameter by roughly 30 cm in length) with a flame-drawn tip. After equilibrating the column each sample was loaded by using a Famos auto sampler (LC Packings) onto the column. A gradient was formed, and peptides were eluted with increasing concentrations of solvent B (97.5% acetonitrile, 0.1% formic acid). As peptides eluted, they were subjected to electrospray ionization and then entered an LTQ Orbitrap Velos Pro ion-trap mass spectrometer (Thermo Fisher Scientific). Peptides were detected, isolated and fragmented to produce a tandem mass spectrum of specific fragment ions for each peptide. For all IP–MS experiments, the following isotype control antibodies were used: rabbit IgG monoclonal (EPR25A) (Abcam, no. ab172730), rabbit IgG polyclonal (Abcam, no. ab37415) and mouse IgG1 κ monoclonal [MOPC-21] (Abcam, no. ab18443). Mass spectrometry experiments that yielded significance for the protein bait over IgG control samples were then included in analyses (all proteins except SOX9).

Peptide sequences (and hence protein identity) were determined by matching the UniProt human protein database (release 2023_01) with the acquired fragmentation pattern using Sequest (Thermo Fisher Scientific)^146^. The database included a reversed version of all the sequences and the data were filtered to between 1% and 2% peptide FDR. Protein quantification was performed using GFY Core Version 3.8 (Harvard University). IP–MS data analysis is described in ref. ^90^.

### PPI analyses

To determine whether the M5 proteins formed a significant interaction network, we compared the logFCs between each ‘bait’ and the other interactors predicted to be in the M5 network and found as a protein in any of the mass spectrometry experiments (943 unique proteins identified across all IP–MS experiments). If the interactors were not pulled down by the bait, the logFC was considered 0. We used the mean logFC across baits as a measure of average connectedness of the network. To assess the statistical significance of these networks, we ran 10,000 permutations by randomly selecting proteins of the same size as the network from the remaining set of 943 uniquely identified proteins. We compared the average logFC of each of these randomly generated networks to create a population curve. We then examined the *z*-scored connectedness of the network. Gene lists used to compare networks were SFARI genes (levels 1, 2, S), ASD risk genes^2,8^, intellectual disability risk genes^91^, NDD risk genes^91^ and developmental disorder enriched, ASD enriched and NDD non-specific^92^.

### gRNA design and CROP-seq cloning

Three genomic RNAs (gRNAs) per target as well as 18 total non-targeting controls (NTC) were designed using the CRISPick database (https://portals.broadinstitute.org/gppx/crispick/public) (Supplementary Table 15). NTCs were both gRNAs with no sequence in the human genome as well as gRNAs targeting random intergenic sequences. Sequences were designed as follows: a 5′ overhang (ATCTTGTGGAAAGGACGAAACACC) and a 3′ overhang (GTTTAAGAGCTATGCTGGAAACAGCATAGCAAGT) that is compatible with the CRISPR droplet sequencing (CROP-seq) vector were added on the outside of the gRNA sequence. A G nucleotide was also incorporated between the 5′ overhang and the gRNA sequence. All oligos were ordered as a pool (Integrated DNA Technologies). The CROP-seq-opti-dsRed vector backbone was obtained from Addgene (Addgene no. 201999) and digested using New England Biolabs’ (NEB) BsmBI enzyme. The oligo pool was diluted at a concentration of 100 µM, PCR amplified, (Q5 Hot Start High-Fidelity 2X Master Mix) gel extracted, and cleaned. The vector and the pooled oligo insert was then assembled (NEBuilder HiFi DNA Assembly Reaction). To validate the insertion was present post assembly, a PCR amplification using the U6 outer forward primer (TTTCCCATGATTCCTTCATATTTGC) and gRNA end reverse primer (AGTACAAGCAAAAAGCAGTGTCTCAA) was run and visualized. All gRNA sequences used for M5 targets and control samples are supplied in Supplementary Tables 14 (targets) and 15 (control samples). The pooled vectors were then transformed into NEB Stable Competent *E. coli* (High Efficiency), and plated at various dilutions on Luria-Bertani medium:ampicillin 1:1,000 plates overnight at 30 °C. Colonies were picked and PCR amplification of the insert was done to insure its presence. All bacterial colonies were then scraped from the plate and grown using 200 ml of 2.5% Luria-Bertani medium + 1:1,000 ampicillin. The broth containing colonies was then shaken at 170 rpm at 30 °C and this was complete once the optical density reached 2.0 nm. DNA was then isolated by use of the Qiagen Maxi Plasmid Kit.

### Lentiviral production

Human embryonic kidney 293T (HEK293T) cells obtained from American Type Culture Collection (lines CRL-3216 and TIB-202) were expanded in DMEM, 10% FBS and 1% penicillin and streptomycin (HEK medium). Then 5 × 10^6^ HEK293T were plated onto each of 2 poly-ornithine (5 µg ml^−1^) coated 10-cm plates for 24 h. Media from both plates was replaced with Opti-MEM before cells from 1 10-cm plate were transduced with a lentiviral vector (pLV-KRAB-dCas9 (Addgene no. 71236) or CROP-seq-opti-dsRed (Addgene no. 201999)) while the other plate of cells was transfected with 15 µg of the pooled plasmid library, 15 µg of psPAX2 and 1.5 µg of VSV-G with lipofectamine 3000 following the manufacturer’s instructions. After 16 h, the Opti-MEM media was replaced with 10 ml of HEK medium, which was collected and replaced every 24 h twice. The HEK medium was then spun down with 12.5 ml of lenti-X concentrator (Takara Bio) and centrifuged at 1,500*g* for 45 min. The supernatant was discarded and the pellet was resuspended in 400 µl of PBS, aliquoted and stored at −80 °C.

### Derivation of NGN2-induced neural cells, transduction and FACS

We derived NGN2-induced hNPCs according to ref. ^100^. A six-well plate was coated with Matrigel (1:50 in Knockout DMEM) for 2 h at 37 °C. A dox-inducible NGN2 iPS cell line, WTC11 (from L. Gan, Weill Cornell, available at Coriell), was seeded at 75,000 cells per cm^2^ in MTeSR+ supplemented with 10 µM of Y-27632. After 24 h, cells were subjected to induction medium (1:100 glutamax, 1:66.7 20% glucose in DMEM:F12, N2 supplement 1:100, doxycycline 2 µg ml^−1^, LDN-193189 200 nM, SB431542 10 µM, XAV939 2 µM). Day 2 involved the induction and selection using the following media: 1:100 glutamax, 1:66.7 20% glucose in DMEM:F12, N2 supplement 1:100, doxycycline 2 µg ml^−1^, LDN-193189 100 nM, SB431542 5 µM, XAV939 1 µM and puromycin 5 µg ml^−1^. Then 24 h postselection, the media was changed fully with NES complete and selection media (DMEM:F12, glutamax 1:50, penicillin and streptomycin 1:100, MEM NEAA 1:100, B-27 without vitamin A 1:50, N2 supplement 1:100, epidermal growth factor 10 ng ml^−1^, basic fibroblast growth factor 10 ng ml^−1^, puromycin 5 µg ml^−1^ and Y-27632 1:1,000). One day later and for the duration of the culture, cells were put on NES complete medium, which is the same as listed above apart from the use of Y-27632 and puromycin. Once hNPCs were generated, 500,000 hNPCs were seeded in every well of a six-well plate with NPC maintenance media (*n* = 6 replicates). To each well, GFP-KRAB-dCas9 (40 µl per well of a six-well plate) plasmid and CROP-dsRed-gRNA (33 µl per well of a six-well plate) were added. Media was replaced daily for 7 days before cells were collected for fluorescence-activated cell sorting (FACS). FACS was done with BD FACSAria, Software FACS Diva v.8.0.2. For FACS, hNPCs were washed with PBS, dissociated into single-cell suspension using Accutase and resuspended in 500 µl of FACS buffer per replicate (7.5% BSA, 0.5 M EDTA, RNase inhibitor 1:200, DAPI 0.5 mg ml^−1^). A population of NPCs that were not transfected with plasmid were used as a negative control to establish FACS gating. Double positive GFP/TdTomato (dCAS9–single-guide RNA double expressing) cells were sorted for downstream single-cell analyses (roughly 30,000 cells per replicate after FACS, roughly 20% of live cell fraction).

### scRNA-seq

Sorted cells then underwent scRNA extraction using the 10X genomics Chromium Next GEM Single Cell 3′ GEM, Library & Gel Bead Kit v.3.1 (PN-1000121). The 10X protocol for library prep was adapted from ref. ^147^. gRNA libraries were sequenced separately from scRNA (GEX) libraries. scRNA and gRNA libraries were aligned to the human genome (GRCh38) with CellRanger (v.7.0.1). Roughly 10,000 aligned cells were recovered per replicate. Cells were filtered to include cells with more than 200 features and percentage of mitochondria less than 10%. Doublets were then removed using DoubletFinder (8,406 doublets, 13.2%). After quality control and doublet filtering before selecting uniquely gRNA positive cells, the median nCounts per cell was 17,071 and the median nGenes was 4,675, from 55,216 singlets (Extended Data Fig. 13c). Single-cell data were then processed using standard best practices for Seurat v.5. Briefly, we log-normalized counts and performed integration using SCTransform followed by clustering. Canonical markers were used to determine cell-type classification including NPC markers SOX2, NES, VIM and HES1 and cNPCs expressing G2 mitotic markers TOP2A, HMGB2, CDK1 and NUSAP1 (Extended Data Fig. 13e). We then assigned gRNAs to cells and filtered cells as follows. BAM files generated from gRNA libraries were analysed as in ref. ^148^ (GitHub, https://github.com/shendurelab/single-cell-ko-screens). In short, a text file of gRNA sequences was supplied as well as the common sequence appearing before the gRNA sequence, and each of these reads was counted from the BAM file and paired to the cells within the scRNA libraries through cell unique molecular identifier matching. Only cells containing gRNA barcodes with a unique molecular identifier count of at least ten were kept in the downstream analysis (Extended Data Fig. 13d). This left a total of 23,672 high-quality singlet cells that had 1 and only 1 gRNA-target assigned (Extended Data Fig. 13d,f). This cut-off has also been previously used^148^ and was determined to be sufficient for significant decrease in expression of the target. All differential analysis was performed on cells that expressed the gRNA of a single target. A standard pseudobulk differential expression using the edgeR (v.3.40.2) Quasi-Likelihood *F* test was performed using the libra package (v.1.0.0) function, run_de(). All differential gene expression compared all cells expressing any control gRNAs with all cells expressing any target gRNAs, above the unique 10 unique-molecular-identifier cut-off (3,163 total cells considered as control cells). The range of cells containing each gene target was 171–2,423, median 775. The proportion of cells containing each unique barcode (summarized by gene target) per replicate is shown in Extended Data Fig. 13g. Each replicate sample, taken from different plate wells was considered one sample for psuedobulking.

### Inclusion and ethics

We have complied with all ethics guidelines and regulations in conducting this research. Informed consent was obtained from all individuals. Participants were recruited at Stanford University, UCLA, NIMH. Ethics oversight is conducted by Stanford University, UCLA and NIMH.

### Reporting summary

Further information on research design is available in the Nature Portfolio Reporting Summary linked to this article.

## Online content

Any methods, additional references, Nature Portfolio reporting summaries, source data, extended data, supplementary information, acknowledgements, peer review information; details of author contributions and competing interests; and statements of data and code availability are available at 10.1038/s41586-025-10047-5.

## Supplementary information

Supplementary Information A guide to Supplementary Tables 1–15 (tables provided separately).

Reporting Summary

Supplementary Tables Supplementary Tables 1–15.

## Data Availability

Processed datasets generated and analysed as a part of this current study are included as Supplementary Tables 1–15. Aligned RNA-seq counts data are available through the Gene Expression Omnibus at accession GSE271853. Owing to participant consent and privacy, we are not able to make raw data public, but they can be made available by the authors on reasonable request. Publicly available data used in this paper were as follows: datasets used to test module preservation: (1) from ref. ^81^, https://www.sciencedirect.com/science/article/pii/S0006322320317029#sec1 (Synapse ID, syn8118403); (2) ref. ^82^; GSE137101; (3) ref. ^83^, GSE46562; (4) ref. ^84^, https://www.ncbi.nlm.nih.gov/pubmed/30617258 (EMBL-EBI ArrayExpress with the accession code E-MTAB-6018) and (5) ref. ^75^, GSE142174. Datasets used for single-cell deconvolution reference to deconvolute days 50–100 at accession no. GSE145122. Datasets used for enrichment testing: (1) SFARI genes https://gene.sfari.org/database/gene-scoring/; (2) ASD risk genes from ref. ^2^, https://pubmed.ncbi.nlm.nih.gov/31981491/ (Supplementary Table 2) and ref. ^8^, https://pubmed.ncbi.nlm.nih.gov/31398340/ (Supplementary Table 3); (3) intellectual disability risk genes and NDD risk genes from ref. ^91^, https://pubmed.ncbi.nlm.nih.gov/33932580/ (Supplementary Table 1) and (4) developmental disorder enriched, ASD enriched and NDD non-specific from ref. ^92^, https://www.nature.com/articles/s41588-022-01104-0 (Supplementary Table 11). Datasets used for genome-wide association study enrichment are available through LDSC: for ASD from ref. ^21^ at https://www.nature.com/articles/s41588-019-0344-8, data available from iPSYCH at https://ipsych.dk/en/research/downloads/2; for schizophrenia from ref. ^129^ at https://www.nature.com/articles/s41588-018-0059-2, data available from the Walters Group at https://walters.psycm.cf.ac.uk/3; for attention-deficit/hyperactivity disorder from ref. ^130^ at https://www.nature.com/articles/s41588-018-0269-7, data through PGC at https://pgc.unc.edu/for-researchers/download-results/; for major depressive disorder from ref. ^131^, https://www.nature.com/articles/s41593-018-0326-7, data available from Edinburgh DataShare at https://datashare.ed.ac.uk/handle/10283/3203 and for bipolar disorder from ref. ^132^, https://www.nature.com/articles/s41588-021-00857-4, data available from PGC at https://pgc.unc.edu/for-researchers/download-results/. The hg38 genome for STAR alignment is available through GENCODE at https://www.gencodegenes.org/human/release_25.html. The Rcistarget databases are available from Stein Aerts laboratory at https://resources.aertslab.org/cistarget/databases/homo_sapiens/hg38/refseq_r80/mc9nr/gene_based/. The UniProt database is available at https://ftp.uniprot.org/pub/databases/uniprot/previous_releases/release-2023_01/.

## Code availability

Code used to analyse and generate plots for this paper is available at Zenodo (10.5281/zenodo.17645341)^149^.
