# Supplementary material for: Developmental convergence and divergence in human stem cell models of autism
Source: Nature. 2026 Jan 29;651(8106):707–19. doi: 10.1038/s41586-025-10047-5 (PMC12999519; doi:10.1038/s41586-025-10047-5)
Supplement: Supplementary file 1 — A guide to Supplementary Tables 1–15 (tables provided separately). [file 41586_2025_10047_MOESM1_ESM.pdf]

nature portfolio

https://doi.org/10.1038/s41586-025-10047-5

# Supplementary information

# Developmental convergence and divergence in human stem cell models of autism

In the format provided by the authors and unedited

Supplementary Information

Supplementary Table 1: RNAseq samples including relevant metadata such as Individual, Cell Line, Differentiation, ASD form, Sex, Differentiation day, RNA seq batch, source tissue, genetic ancestry PCs 1 and 2, and Sequencing PCs 1- 15.

Supplementary Table 2: WGS samples. Additional lines were QC'd in Khan et al. 2022

Supplementary Table 3: DE results from dreamlet. Each sheet is an ASD form at each time of differentiation.

Supplementary Table 4: Day 25 overlapping genes - GO terms. Selected data plotted in Figure 2d.

Supplementary Table 5: Gene set enrichment of DEGs. Calculated from fgsea. Selected data plotted in Figure 3a

Supplementary Table 6: ICA Gene set enrichment. Calculated from fgsea. Selected data plotted in Extended Data Figure 9b.

Supplementary Table 7: WGCNA modules from day 25. Includes genes in each module and KMEM score for each gene in each module.

Supplementary Table 8: WGCNA GO terms. Gene ontology terms, associated genes and enrichment statistics for each module.

Supplementary Table 9: WGCNA TF binding enrichment. Rcistarget results for enriched DNA binding proteins upstream of genes in each module.

Supplementary Table 10: M5 PPI GO term enrichment. Gene ontology enrichment of M5 PPI.

Supplementary Table 11: M5 proteins IP MS results showing each individual replicate and statistics of protein interactions.

Supplementary Table 12: DE targets of M5 TFs. Libra (edgeR) output for each CRISPRi target.

Supplementary Table 13: GSEA results of M5 CRISPRi. Calculated from fgsea. Selected data plotted in Extended Data Figure 15.

Supplementary Table 14: gRNA sequences. All gRNA sequences for M5 target genes.

Supplementary Table 15: gRNA sequences. All gRNA sequences used as controls. Includes non-targeting controls and random sequences
