# Supplementary material for: Developmental convergence and divergence in human stem cell models of autism
Source: Nature. 2026 Jan 29;651(8106):707–19. doi: 10.1038/s41586-025-10047-5 (PMC12999519; doi:10.1038/s41586-025-10047-5)
Supplement: Supplementary file 2 — Reporting Summary [file 41586_2025_10047_MOESM2_ESM.pdf]

nature portfolio

Corresponding author(s): Dan Geschwind, dhg@mednet.ucla.edu and Sergiu Pasca, spasca@stanford.edu

Last updated by author(s): Nov 19, 2025

Reporting Summary

Nature Portfolio wishes to improve the reproducibility of the work that we publish. This form provides structure for consistency and transparency in reporting. For further information on Nature Portfolio policies, see our Editorial Policies and the Editorial Policy Checklist.

Statistics

For all statistical analyses, confirm that the following items are present in the figure legend, table legend, main text, or Methods section.

n/a	Confirmed	
☐	☒	The exact sample size (*n*) for each experimental group/condition, given as a discrete number and unit of measurement
☐	☒	A statement on whether measurements were taken from distinct samples or whether the same sample was measured repeatedly
☐	☒	The statistical test(s) used AND whether they are one- or two-sided<br>*Only common tests should be described solely by name; describe more complex techniques in the Methods section.*
☐	☒	A description of all covariates tested
☐	☒	A description of any assumptions or corrections, such as tests of normality and adjustment for multiple comparisons
☐	☒	A full description of the statistical parameters including central tendency (e.g. means) or other basic estimates (e.g. regression coefficient) AND variation (e.g. standard deviation) or associated estimates of uncertainty (e.g. confidence intervals)
☐	☒	For null hypothesis testing, the test statistic (e.g. *F*, *t*, *r*) with confidence intervals, effect sizes, degrees of freedom and *P* value noted<br>*Give P values as exact values whenever suitable.*
☒	☐	For Bayesian analysis, information on the choice of priors and Markov chain Monte Carlo settings
☒	☐	For hierarchical and complex designs, identification of the appropriate level for tests and full reporting of outcomes
☐	☒	Estimates of effect sizes (e.g. Cohen's *d*, Pearson's *r*), indicating how they were calculated

Our web collection on statistics for biologists contains articles on many of the points above.

Software and code

Policy information about availability of computer code

Data collection	BD FACSAria, Software FACS Diva 8.0.2.
Data analysis	RcisTarget (v1.6.0). Dapple (v0.19). CellRanger (7.0.1). BisqueRNA package (v.1.0.5) . car package (v3.0.11). multicomp package (v1.4.18). nlme package (v3.1.152) . dendextend package (v1.14.0) . fossil package (v0.4.0) . pvclust package (v2.2.0) . ComplexHeatmap package (v2.9.3) . EWCE package (v.1.0.1) . LDscore regression (v1.0.0) . clusterProfiler (v4.0.5) . (cqn v1.36.0) . MineICA package (v1.30.0) . edgeR package (v3.26.8) and v(3.40.2) . variancePartition package (v1.20.0) . metafor (v4.6.0) . fgsea package (v1.10.1) and fgsea package (v1.3.0) . WGCNA package (v1.70.3). STAR (v.2.5.2b) . rsem (v1.3.0) . GATK (v3.3) . HapMap3.3. PLINK (1.09). bwa-mem (v0.7.17) . bamtools (v2.5.1) . Picard MarkDuplicates tool (v2.5.0). DELLY (v0.8.7) . Seuratv4 and Seuratv5. VQSR (v3.3) . IGV (v2.9.4) . Samtools (v1.9) . libra (1.0.0) . Code has been made available at 10.5281/zenodo.17645341

For manuscripts utilizing custom algorithms or software that are central to the research but not yet described in published literature, software must be made available to editors and reviewers. We strongly encourage code deposition in a community repository (e.g. GitHub). See the Nature Portfolio guidelines for submitting code & software for further information.

Data

Policy information about availability of data

All manuscripts must include a data availability statement. This statement should provide the following information, where applicable:

- Accession codes, unique identifiers, or web links for publicly available datasets
- A description of any restrictions on data availability
- For clinical datasets or third party data, please ensure that the statement adheres to our policy

We have detailed the location of data generated in this study and both datasets and databases used in this study, as well as descriptions of restrictions on raw data in the data availability section as follows: "Processed datasets generated and analyzed as a part of this current study are included as supplementary tables. Aligned RNA-seq counts data is available on GEO: GSE271853. Due to patient consent and privacy, we are not able to make raw data public, but it can be made available by the authors upon reasonable request. Publically Available data used in this paper: Datasets used to test module preservation: 1) Adhya et al: https://www.sciencedirect.com/science/article/pii/S0006322320317029#sec1 (Synapse ID: syn8118403) 2) Flaherty et al; GSE137101 Lin et al: GSE46562 4) Schafer et al: https://www.ncbi.nlm.nih.gov/pubmed/30617258 (EMBL-EBI ArrayExpress with the accession code: E-MTAB-6018) 5) Urresti et al: GSE142174 Dataset used for single cell deconvolution reference to deconvolute days 50-100: GSE145122 Datasets used for enrichment testing: 1) SFARI genes https://gene.sfari.org/database/gene-scoring/ 2) ASD risk genes Satterstrom et al: https://pubmed.ncbi.nlm.nih.gov/31981491/ (Table S2) and Ruzzo et al: https://pubmed.ncbi.nlm.nih.gov/31398340/ (Table S3) 3) ID risk genes and NDD risk genes Leblond et al: https://pubmed.ncbi.nlm.nih.gov/33932580/ (Table S1) 4) DD-enriched, ASD-enriched and NDD-nonspecific from Fu et al: https://www.nature.com/articles/s41588-022-01104-0 (Table S11). Datasets used for GWAS enrichment via LDSC 1) ASD: https://www.nature.com/articles/s41588-019-0344-8 (data at https://ipsych.dk/en/research/downloads/) 2) SCZ: https://www.nature.com/articles/s41588-018-0059-2 (data at https://walters.psycm.cf.ac.uk/) 3) ADHD: https://www.nature.com/articles/s41588-018-0269-7 4) (data at https://pgc.unc.edu/for-researchers/download-results/) MDD: https://www.nature.com/articles/s41593-018-0326-7 (data at https://datashare.ed.ac.uk/handle/10283/3203) 5) BD: https://www.nature.com/articles/s41588-021-00857-4 (data at https://pgc.unc.edu/for-researchers/download-results/). hg38 genome for STAR alignment: https://www.gencodegenes.org/human/release_25.html For Rcistarget databases: https://resources.aertslab.org/cistarget/databases/homo_sapiens/hg38/refseq_r80/mc9nr/gene_based/ For Uniprot database https://ftp.uniprot.org/pub/databases/uniprot/previous_releases/release-2023_01/"

Research involving human participants, their data, or biological material

Policy information about studies with human participants or human data. See also policy information about sex, gender (identity/presentation), and sexual orientation and race, ethnicity and racism.

Reporting on sex and gender	Sex was collected from self reporting and confirmed via whole genome sequencing. Sex was included as a co-variate in the model to determine differential gene expression, WGCNA, and ICA. Supplementary table 1 includes all samples used and their biological sex as well as other covariates.
Reporting on race, ethnicity, or other socially relevant groupings	The first two principal components of genetic ancestry, calculated from whole genome sequencing, were used as covariates in the model to determine different gene expression, WGCNA, and ICA. Supplementary table 1 includes the first two genetic ancestry PCs.
Population characteristics	18 patients with 22qll.2 deletion, 11 patients with Idiopathic ASD, 4 patients with 22q13.3 deletion, 3 patients with 15q13.3 deletion, 4 patients with 16pll.2 duplication, 4 patients with 16pll.2 deletion, 2 patients with Timothy Syndrome, 2 patients with PCDH19 related disorder and 1 patient with SHANK3 mutation and 25 controls as shown in Figure la.
Recruitment	Informed consent was obtained from all individuals. Subjects were recruited at Standford University, UCLA, NIMH
Ethics oversight	Stanford University, UCLA, NIMH

Note that full information on the approval of the study protocol must also be provided in the manuscript.

Field-specific reporting

Please select the one below that is the best fit for your research. If you are not sure, read the appropriate sections before making your selection.

☒ Life sciences ☐ Behavioural & social sciences ☐ Ecological, evolutionary & environmental sciences

For a reference copy of the document with all sections, see nature.com/documents/nr-reporting-summary-flat.pdf

Life sciences study design

All studies must disclose on these points even when the disclosure is negative.

Sample size	Sample size was not pre-calculated due to the nature of patient-derived organoid work. We aimed to maximize the sample size through patient recruitment. Our starting sample sizes have a wide range depending on the specific mutation, however, our sample size is the largest in organoid work to date, with a starting total of 96 lines from 74 individuals resulting in 70 lines from 55 individuals after rigorous data quality control. Sample sizes were chosen based on the number of patient derived lines that were available. While overall the co-hort is the largest patient-derived iPSC organoid work to date, the sample size varies from very large (i.e. 22q11.2del starting from 24 derived iPSCs from 18 subjects) to small (i.e. SHANK3 2 lines from 1 subject). Because of this, we emphasize convergent findings across genetically defined forms of ASD, which we are highly powered to do.

Data exclusions

Data exclusions are described in detail in the methods section of this paper, briefly, we conducted whole genome sequencing and dropped lines and individuals with genomic abnormalities or where the mutation was not confirmed. We also dropped RNA-sequencing samples if they did not pass RNA-seq QC metrics (duplication levels, intergenic mapping, or low mRNA levels). All RNA-seq samples that passed QC are listed in Supplementary Table l.

Replication

Our design included both within individual replication with different iPSC lines derived from the same individual as well as within line replication with different differentiations derived from the same line. We show correlations for replication at both levels in figure 1 and we find high levels of reproducibility, another strength of our study.

Randomization

Because we examined patient-derived cell lines from individuals with various genetic forms of ASD (and Idiopathic patients as well as Controls), randomization was not a part of this study. However, co-variates were controlled in all statistical analyses. Allocation was random into RNA-sequencing batches. Technical and biological co-variates include Individual, Cell line, Induction, Sex, Ethnicity PC 1 and 2, and Sequencing PCS 1-15, derived directly from sequencing bam files.

Blinding

RNA-sequencing libraries were created by blinded individuals. RNA-sequencing experimentation is the only relevant experiment involving different groups, and therefore all experiments were done with blinding. For CRISPRi experiments all of the replicates receive the entire library of controls and gRNA targets, so there are no differences between replicates/no groups to be blinded against.

Reporting for specific materials, systems and methods

We require information from authors about some types of materials, experimental systems and methods used in many studies. Here, indicate whether each material, system or method listed is relevant to your study. If you are not sure if a list item applies to your research, read the appropriate section before selecting a response.

Materials & experimental systems

n/a	Involved in the study
☐	☒ Antibodies
☐	☒ Eukaryotic cell lines
☒	☐ Palaeontology and archaeology
☒	☐ Animals and other organisms
☒	☐ Clinical data
☒	☐ Dual use research of concern
☒	☐ Plants

Methods

n/a	Involved in the study
☒	☐ ChIP-seq
☐	☒ Flow cytometry
☒	☐ MRI-based neuroimaging

Antibodies

Antibodies used

RELA (Active motif 40916), TPS3 (Abeam ab26), CNOT6 (CST #1341S), TBP (CST #8S15), POLR2A (PTG 20655-1-AP), SOX9 (CST #82630), SMARCBl (CST #91735), SMARCA4 (Abeam ab110641), and EP300 (Abeam ab275378) . The following description from the Methods section outlines concentration and use of antibodies "We tested antibodies against RELA (Active motif 40916), TP53 (Abcam ab26), CNOT6 (CST #13415), TBP (CST #8515), POLR2A (PTG 20655-1-AP), SOX9 (CST #82630), SMARCB1 (CST #91735), SMARCA4 (Abcam ab110641), and EP300 (Abcam ab275378). All antibodies were run at 1:1000 concentrations. " As well as the following section detailing secondary antibody use "After 3 × 10-min washes in TBST, blots were incubated with horseradish peroxidase (HRP)–conjugated secondary antibodies for 45 min and again washed three times in TBST. The following secondary antibodies were used: anti-mouse IgG HRP-linked (NA9310V, Sigma-Aldrich) and anti-rabbit IgG HRP-linked (GENA934, Sigma-Aldrich) at a concentration of 1:5000. "

Validation

Antibodies that generated western blot bands at the appropriate size were selected for continued mass spectrometry (MS) analysis. Secondary validation consists of IP confirmed enrichment of bait protein peptides pulled down by the validated antibody, consistent with the band detected in the western blot. For all IP-MS experiments, the following isotype control antibodies were used: rabbit IgG monoclonal [EPR25A] (Abcam, Cat# ab172730), rabbit IgG polyclonal (Abcam, Cat# ab37415), and mouse IgG1 κ monoclonal [MOPC-21] (Abcam, Cat# ab18443). The following statements are from the manufacturers: RELA Active motif 40916 - Validated by Active Motif for western blot (WB) and CHIP. Not validated in our hands and dropped from analysis. TPS3 (Abcam ab26) - Validated by Abcam for WB, IP, ICC, IF. KO validated for confirmed specificty. NOT validated in our hands and dropped from analysis. CNOT6 (CST #1341S) - Validated by CST for WB, NOT validated in our hands and dropped from analysis. TBP (CST #8S15) - Validated by CST for WB, confirmed validation in our hands through WB and IP MS. POLR2A (PTG 20655-1-AP) - Validated by PTG for WB, IHC, ELISA. Validated by us for WB and IP MS. SOX9 (CST #82630) - Validated by CST for WB, IHC. Validated by us for WB, not validated for IP MS. SMARCB1 (CST #91735) - Validated by CST for WB, IP, CHIP, IHC, C&R. Validated by us by WB and IP MS. SMARCA4 (Abcam ab110641) - validated by Abcam for WB, Flow Cytometry, IP, IHC, IF. Validated with KO validation for confirmed specificity. Validated by us by WB, IP MS. EP300 (Abcam ab275378) - validated by Abcam for WB, Flow Cytometry, IP, CHIP, IF. Validated with KO for confirmed specificity. Validated by us by WB, IP MS.

Eukaryotic cell lines

Policy information about cell lines and Sex and Gender in Research

Cell line source(s)

Human fibroblast cells were reprogrammed using the Sendai virus-based CytoTune iPS 2.0 kit CytoTune 2.0, lnvitrogen, A16517). hiPS cell lines from 11 idiopathic ASD individuals were obtained from Coriell. HEK293T were used only for lentiviral

	production. and were obtained from ATCC (CRL-3216 and TIB-202). The iPSC line, WTC11 was used for CRISPRi experiments is from Dr. Li Gan (Weill Cornell) and is available at https://catalog.coriell.org/0/Sections/Search/Sample_Detail.aspx?Ref=GM25256&Product=CC
Authentication	Whole Genome Sequencing was done to authenticate each patient line and differentiation.
Mycoplasma contamination	Cultures were regularly tested for mycoplasma and maintained mycoplasma free.
Commonly misidentified lines (See ICLAC register)	No commonly misidentified cell lines were used in this study

Plants

Seed stocks	*Report on the source of all seed stocks or other plant material used. If applicable, state the seed stock centre and catalogue number. If plant specimens were collected from the field, describe the collection location, date and sampling procedures.*
Novel plant genotypes	*Describe the methods by which all novel plant genotypes were produced. This includes those generated by transgenic approaches, gene editing, chemical/radiation-based mutagenesis and hybridization. For transgenic lines, describe the transformation method, the number of independent lines analyzed and the generation upon which experiments were performed. For gene-edited lines, describe the editor used, the endogenous sequence targeted for editing, the targeting guide RNA sequence (if applicable) and how the editor was applied.*
Authentication	*Describe any authentication procedures for each seed stock used or novel genotype generated. Describe any experiments used to assess the effect of a mutation and, where applicable, how potential secondary effects (e.g. second site T-DNA insertions, mosiacism, off-target gene editing) were examined.*

Flow Cytometry

Plots

Confirm that:

☒ The axis labels state the marker and fluorochrome used (e.g. CD4-FITC).

☒ The axis scales are clearly visible. Include numbers along axes only for bottom left plot of group (a 'group' is an analysis of identical markers).

☒ All plots are contour plots with outliers or pseudocolor plots.

☒ A numerical value for number of cells or percentage (with statistics) is provided.

Methodology

Sample preparation	Sample preparation is described in detail in the methods section. Briefly, hNPCs were washed with PBS, dissociated into single cell suspension using accutase and resuspended in 500 ul FACS buffer.
Instrument	BD FACSAria
Software	BD FACSDiva 8.0.2
Cell population abundance	double positive cells for GFP and dsRed were 21-23 % of the live cell fraction.
Gating strategy	A population of NPCs that was not transfected with plasmid were used as a negative control to establish FACS gating.

☒ Tick this box to confirm that a figure exemplifying the gating strategy is provided in the Supplementary Information.
